# Configurational entropy of a finite number of dumbbells close to a wall

**DOI:** 10.1140/epje/s10189-022-00160-y

**Published:** 2022-01-24

**Authors:** Markus Hütter

**Affiliations:** Department of Mechanical Engineering, Polymer Technology, Eindhoven University of Technology, PO Box 513, 5600 MB Eindhoven, The Netherlands

## Abstract

**Abstract:**

The effect of confinement on the conformation of *N* dumbbells in *D* dimensions close to a non-interacting and rigid flat wall is examined. Using statistical mechanics and numerical calculations, the partition coefficient and the confinement-induced change in the configurational entropy are calculated as a function of the conformation tensor ${\varvec{c}}$ and of the distance of the dumbbells from the wall. Analytical predictions and numerical results for $D=1$ concerning the behavior close to the limiting cases (onset of and saturation of confinement) agree favorably; in one case where an analytical prediction has not been achieved, a thorough numerical study establishes the limiting behavior nevertheless. Beyond these limiting cases, the overall behavior of the partition coefficient and the configurational entropy has been examined as well in detail, for various choices of the parameters. Furthermore, it is shown that the effect of confinement for $D>1$ is captured entirely by the partition coefficient determined for $D=1$. In general, the average extension of the dumbbells in the direction perpendicular to the wall is decreased the closer the dumbbells are to the wall. Also, the decay of the partition coefficient with increasing extension of the dumbbells becomes steeper, i.e., more localized, the higher the number of dumbbells *N*. Finally, it is discussed under what conditions these results can be used also for the case of slab- (i.e., slit-) confinement.

**Graphical abstract:**

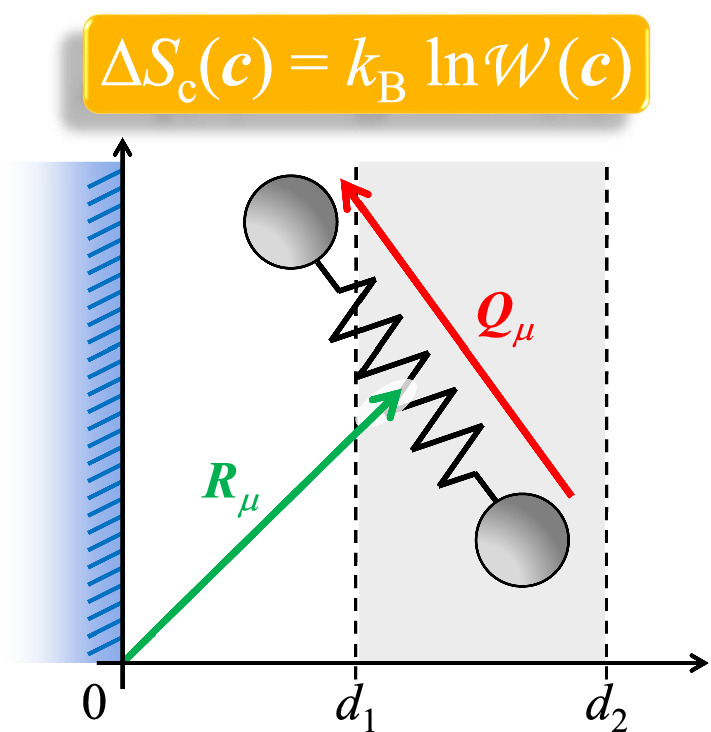

## Introduction

The static and dynamic properties of polymers are affected by the presence of nearby obstacles, since the possible conformations of the polymer chains are reduced substantially, see [1, 2]. There are several fields in which this is of practical relevance, e.g. in size exclusion chromatography, interaction of biological macromolecules with membrane surfaces, polymer-assisted flocculation and stabilization of colloids, and surface modification and coatings [1]. Throughout this paper, only obstacles will be considered that are non-interacting and rigid (i.e., not flexible).

Theory and simulations have been used extensively to examine how the conformations of polymer chains are affected by obstacles. A very prominent technique is based on the notion that the polymer chain is represented by a random walk, which in turn is studied in terms of a diffusion equation, according to Chandrasekhar [3], where the obstacles (i.e., the confinement by walls) are taken into account by appropriate (absorption) boundary conditions [4]. There are many examples where this approach has been followed, see e.g. [1, 5–10]. Another technique that has been usedextensively is that of Monte Carlo simulations, both on- [11–16] and off-lattice [17, 18]; for a review on lattice models for polymers close to interfaces, the reader is referred to [19]. Furthermore, also scaling arguments and the blob concept have been used to study confinement effects [20]. While the techniques mentioned so far address static properties, the effects of confinement on the dynamics of polymer chains have been studied by molecular dynamics simulations, e.g. [21, 22].

Different types of confinement have been studied by theory and simulations, e.g. polymers close to a single flat wall [8, 10, 21], polymers between two parallel walls (so-called slab- or slit-geometries) [11–17, 20] and polymers in thin-films [22], in spherical cavities [18], and in capillaries/cylindrical cavities [13, 20, 22]. Experimental studies on confinement effects typically are concerned with planar constraints such as flat surfaces and (ultra-)thin films, see e.g. [23].

A quantity frequently examined in relation to confinement is the so-called partition (or distribution) coefficient, which is the ratio of the partition functions of the polymer chains with and without the confinement, respectively [1, 2, 5–7]; this coefficient grows (and eventually approaches unity) as the strength of the confinement effect on the polymer chains is weakened, e.g., by increasing the spacing between the confining walls. For some specific cases, the partition coefficient—which is closely related to the change in free energy due to confinement—has been calculated, e.g., for polymer chains in a sphere, cylinder, or slab [2, 5, 6, 14–16, 20]. Concerning the effect of confinement on the conformation of polymer chains, a prototypical case to look at is that of flat surfaces: It is generally accepted that the polymer coil is compressed in the direction perpendicular to the surface; in contrast, it depends on system specifics and the proximity to the wall whether and to what extent the coil extension in the direction parallel to the surface is increased the stronger the confinement [10, 12–14, 16, 17, 22, 23].

The dynamics of polymer liquids when exposed to deformation can be conveniently described in terms of a microstructural variable, e.g., in terms of the conformation tensor [24], and thermodynamic approaches have been employed to derive two-scale models, see e.g. [25, 26]. Recently, a conformation tensor-based approach has been used to formulate models for viscoelasticity with thermal fluctuations [27–29], in which the configurational entropy and free energy play a key role. It has been shown how such models, incl. fluctuations, can be derived from an underlying description of a finite number *N* of dumbbells [30], particularly emphasizing the finite-size (*N*) effects in the configurational entropy and free energy. When applying a conformation-tensor approach to a polymeric liquid flowing under narrow confinement, e.g. in a capillary, some portions of the liquid are close to the wall, and therefore the configurational entropy should reflect these confinement effects. This is what has been addressed also in the comprehensive work of Mavrantzas and Beris [8–10]. In their approach, confinement effects on the polymer conformations are taken into account by studying the diffusion equation for the polymer random walk, with appropriate boundary conditions (see above). However, both in their work and in the rest of the literature, to the best of our knowledge, neither the configurational entropy nor the partition coefficient are studied explicitly as functions of the conformation tensor. For modeling polymer liquids at small scales under confinement, these two quantities are of significant interest, not only for static properties but also when building dynamic models along a thermodynamics route [25, 26]. Therefore, in this paper, we will depart from our earlier finite-*N* calculation of the configurational entropy [30], and extend it to include the effects of confinement due to a nearby flat wall, by a statistical mechanics calculation.

The paper is organized as follows: After introducing notation and defining the task properly in Sect. 2, general arguments about the configurational partition function of confined dumbbells (as a reduced description of polymer chains) are presented in Sect. 3. Thereafter, the cases of the spatial dimension being equal to unity ($D=1$) or larger than unity ($D > 1$) are discussed in detail, in Sects. 4 and  5, respectively. Finally, the results are discussed and conclusions are drawn in Sect. 6.

## Problem definition

### Notation

Throughout this paper, the following notation will be used: All summations are spelled out, i.e., no Einstein summation-convention is used for repeated indices. Latin indices are used to denote Cartesian components, while Greek indices are used for enumerating the dumbbells. The symbol $\cdot$ denotes a contraction of one pair of indices. The Kronecker delta is given as $\delta _{ij}$, and the Dirac delta-function as $\delta (\ldots )$. The dyadic product of two vectors ${\varvec{v}}_1$ and ${\varvec{v}}_2$ is written as ${\varvec{v}}_1 {\varvec{v}}_2$.

### Characterization of dumbbells, confinement

Let us consider *N* dumbbells in *D* dimensions, where the positions of the two ends of the dumbbells are denoted by the *D*-vectors ${\varvec{x}}_\mu$ and ${\varvec{y}}_\mu$ ($\mu = 1, \ldots , N$). For each dumbbell, one can define the center-of-mass position ${\varvec{R}}_\mu$ and connector vector ${\varvec{Q}}_\mu$,

1 $$\begin{aligned} {\varvec{R}}_\mu= & {} \frac{1}{2} \left( {\varvec{x}}_\mu + {\varvec{y}}_\mu \right) \, , \end{aligned}$$

2 $$\begin{aligned} {\varvec{Q}}_\mu= & {} {\varvec{y}}_\mu - {\varvec{x}}_\mu \, . \end{aligned}$$

The dumbbells are fully head-tail symmetric. The instantaneous conformation tensor for the assembly of all dumbbells can be written as

3 $$\begin{aligned} \hat{{\varvec{c}}}= \frac{1}{N} \sum ^N_{\mu =1}{\varvec{Q}}_\mu {\varvec{Q}}_\mu \, . \end{aligned}$$

**Fig. 1:**
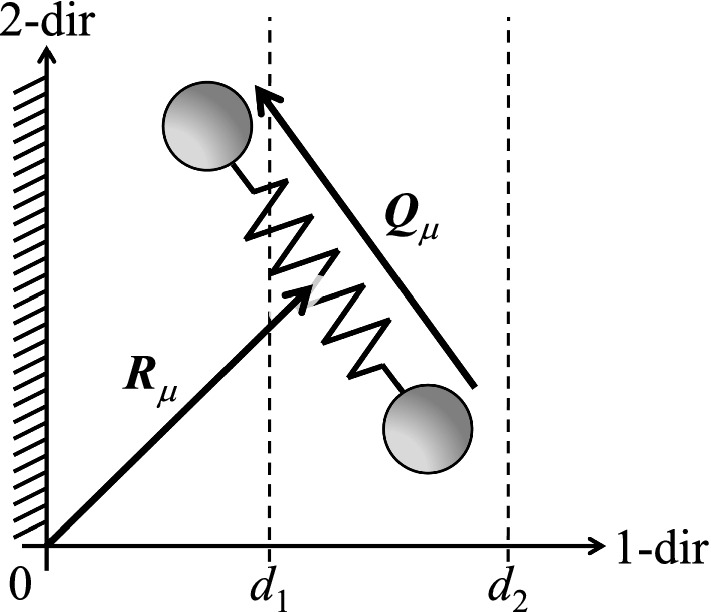
Illustration of a dumbbell in 2 dimensions. Symbols are explained in the text

Consider that all beads are to the right of a flat and hard impenetrable wall with surface-normal in the 1-direction (see also Fig. 1):

4 $$\begin{aligned} x_{\mu ,1}\ge & {} 0 \, , \end{aligned}$$

5 $$\begin{aligned} y_{\mu ,1}\ge & {} 0 \, . \end{aligned}$$

In the following, we are not only interested in all beads being on one and the same side of the wall; in addition, we require that the center-of-mass position of each dumbbell is in a certain slab parallel to the wall (see Fig. 1):

6 $$\begin{aligned} 0 \le d_1\le R_{\mu ,1} \le d_2\, . \end{aligned}$$

This additional requirement is relevant when carrying out finite-element calculations of the liquid flow past the wall: Having in mind a spatial discretization in the vicinity of the wall, a volume element positioned at a finite distance from the wall corresponds to $0< d_1 (< d_2)$, while the volume element adjacent to the wall is represented by $0 = d_1 (< d_2)$. In order to be able to carry out spatially inhomogeneous calculations, with volume elements at various distances from the wall, it is necessary to know the configurational entropy in each of the volume elements (i.e., in each slab). Due to $R_{\mu ,1} \le d_2$, no bead may be further away from the wall than $2d_2$, since otherwise one of the two conditions Eqs. (4) and  (5) would be violated. The above three conditions (4)–(6) can thus be written as the following parametrization of the domain of accessible positions (all but the 1-direction have no restrictions),

7 $$\begin{aligned} 0\le & {} x_{\mu ,1} \le 2d_2\, , \end{aligned}$$

8 $$\begin{aligned} \max \left( 2 d_1- x_{\mu ,1},0\right)\le & {} y_{\mu ,1} \le 2d_2- x_{\mu ,1} \, . \end{aligned}$$

**Fig. 2:**
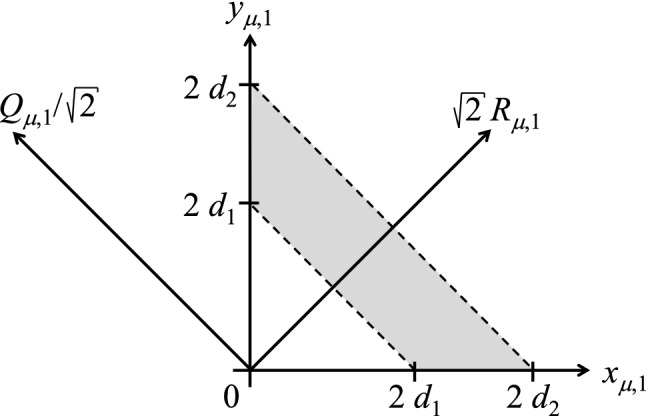
Illustration of the change of variables. The gray-shaded area denotes the admissible part of the configuration space

For practical purposes, it is convenient to parametrize the domain of accessible states as (see Fig. 2)

9 $$\begin{aligned} 0\le & {} d_1\le R_{\mu ,1} \le d_2\, , \end{aligned}$$

10 $$\begin{aligned} -2 R_{\mu ,1}\le & {} Q_{\mu ,1} \le 2 R_{\mu ,1} \, . \end{aligned}$$

It is noted that the determinant for the transformation of variables from $\{{\varvec{x}}_\mu ,{\varvec{y}}_\mu \}$ to $\{{\varvec{R}}_\mu ,{\varvec{Q}}_\mu \}$ is equal to unity. The space of admissible $\{{\varvec{R}}_\mu \}$ and $\{{\varvec{Q}}_\mu \}$ will be called configuration space.

### Helmholtz free energy from statistical mechanics

Given the conformation tensor ${\varvec{c}}$, the Helmholtz free energy $\Psi$ is given by $\Psi = -k_\mathrm{{B}} T\ln Z$, with the canonical partition-function for a finite number *N* of dumbbells [30]

11 $$\begin{aligned} Z({\varvec{c}}) = \int _{\varOmega _R} \int _{\varOmega _Q} e^{-\varPhi /(k_\mathrm{{B}} T)} \, \delta ^{(K)}\left( \hat{{\varvec{c}}}-{\varvec{c}}\right) \, d^{D N}\!Q \, d^{D N}\!R \, , \nonumber \\ \end{aligned}$$

where $\varPhi$ denotes the energy for a certain conformation of the dumbbells. The *K*-dimensional Dirac $\delta$-function makes sure that only those states in configuration space are accounted for that are compatible with the conformation tensor ${\varvec{c}}$. Since $\hat{{\varvec{c}}}$ is symmetric by definition, see Eq. (3), only $K = D(D + 1)/2$ independent conditions are needed (instead of $D^2$); no more conditions are required for properly restricting the integration in $\{{\varvec{Q}}_\mu \}$-space [30]. The integration domains in Eq. (11) are given by

12 $$\begin{aligned} \varOmega _R= & {} \otimes ^{N} \left( [d_1, d_2] \otimes \left( \otimes ^{(D-1)} [l_1, l_2] \right) \right) \, , \end{aligned}$$

13 $$\begin{aligned} \varOmega _Q= & {} \otimes ^{N} \left( [-2 R_{\mu ,1}, 2 R_{\mu ,1}] \otimes {\mathbb R}^{(D-1)} \right) \, , \end{aligned}$$

i.e., for each bead we must have $d_1\le R_{\mu ,1} \le d_2$ and $l_1\le R_{\mu ,i} \le l_2$ for $1 < i \le D$, while $-2 R_{\mu ,1} \le Q_{\mu ,1} \le 2 R_{\mu ,1}$ and all other components of ${\varvec{Q}}_\mu$ are not restricted. It is pointed out that $\delta ^{(K)}$ is actually a $\delta$-function in ${\varvec{c}}$-space, which means $\int \delta ^{(K)}(\hat{{\varvec{c}}}-{\varvec{c}}) d^{K}c = 1$ [30].

If we restrict our attention to cases where $\varPhi$ depends on the dumbbell conformations only by way of the instantaneous conformation tensor $\hat{{\varvec{c}}}$ (see [30] for examples), one can write

14 $$\begin{aligned} Z({\varvec{c}}) = e^{-\varPhi ({\varvec{c}})/(k_\mathrm{{B}} T)} \varGamma ({\varvec{c}}) \, , \end{aligned}$$

with

15 $$\begin{aligned} \varGamma ({\varvec{c}}) = \int _{\varOmega _R} \int _{\varOmega _Q} \, \delta ^{(K)}\left( \hat{{\varvec{c}}}-{\varvec{c}}\right) \, d^{D N}\!Q \, d^{D N}\!R \, . \end{aligned}$$

Considering the integrand of the *R*-integral, the only *R*-dependence is in the integration domain $\varOmega _Q$, and therefore all integrations other than $R_{\mu ,1}$ can be performed, leading to $\varGamma = \left( l_2- l_1\right) ^{N(D-1)} \mathcal {G}$, with

16 $$\begin{aligned} \mathcal {G}= \int _{\varOmega _{\bar{R}}^{{\varvec{d}}}} \int _{\varOmega _Q^{\{R_{\mu ,1}\}}} \, \delta ^{(K)}\left( \hat{{\varvec{c}}}-{\varvec{c}}\right) \, d^{D N}\!Q \, d^{N}\!R \, , \end{aligned}$$

where

17 $$\begin{aligned} \varOmega _{\bar{R}}^{{\varvec{d}}}= & {} \otimes ^{N} [d_1, d_2] \, . \end{aligned}$$

With all this, the free energy becomes

18 $$\begin{aligned} \Psi = \varPhi - k_\mathrm{{B}} TN (D-1) \ln (l_2-l_1) - k_\mathrm{{B}} T\ln \mathcal {G}\, , \end{aligned}$$

where only the first and the third contributions on the right-hand side depend on the conformation tensor ${\varvec{c}}$.

The effect of the confinement on the conformations of the dumbbells is encoded in $\mathcal {G}$, which is related to the configurational entropy (omitting the ${\varvec{c}}$-independent additive contribution for simplicity),

19 $$\begin{aligned} S_\mathrm{c} = k_\mathrm{B} \ln \mathcal {G}\, . \end{aligned}$$

In order to emphasize the effect of confinement, it is convenient to split off the solution $\mathcal {G}_0$ in the absence of confinement, which is given as $\mathcal {G}_0 = (d_2-d_1)^{N} (\det {\varvec{c}})^{(N-D-1)/2}$, according to [30]. Using $\mathcal {G}_0$, one can define the quantity

20 $$\begin{aligned} \mathcal {W}= \frac{\mathcal {G}}{\mathcal {G}_0} \, , \end{aligned}$$

where the symbol $\mathcal {W}$ is used to emphasize that the confinement is caused by a wall. The definition in Eq. (20) is analogous to the partition coefficient referred to in Sect. 1, however in our case now this is a function of the conformation tensor ${\varvec{c}}$; despite this difference, we shall call also $\mathcal {W}$ the “partition coefficient”. Notably, the change in configurational entropy (Eq. 19) due to confinement can be expressed as

21 $$\begin{aligned} \varDelta S_\mathrm{c} \equiv S_\mathrm{c} - S_\mathrm{c,0} = k_\mathrm{B} \ln \mathcal {W}\, , \end{aligned}$$

where we have made use of the definition in Eq. (20). It is noted that the indistinguishability of the identical particles per dumbbell and of the identical dumbbells is not taken into account explicitly, since that would amount to the same multiplicative factor to both $\mathcal {G}$ and $\mathcal {G}_0$, which leaves the partition coefficient $\mathcal {W}$ invariant (see Eq. (20)).

While there is no physical interaction between the dumbbells, they become coupled when calculating the partition function (i.e., the number of microstates) for given conformation tensor ${\varvec{c}}$, because the latter depends on the extension of all dumbbells simultaneously. Therefore, one can anticipate a non-trivial dependence of the partition function on the number of dumbbells *N* despite the absence of physical interaction.

## Scaling behavior of the partition function

In our earlier work, basically two distinct procedures have been presented for calculating the partition function $\mathcal {G}$, in the absence of confinement: one based on scaling arguments and another one based on a differential equation [30]. In order to account for confinement, one could choose either of the two procedures in principle, and it can be shown by explicit (although lengthy in the case of the differential equation) calculations that the results are identical. Since the approach using the scaling argument is more compact and straightforward, we follow only this route here.

To proceed, it is chosen to describe the $\{{\varvec{Q}}_\mu \}$-space in terms of the *N*-dimensional vectors $\mathbf {X}_i$, $1 \le i \le D$, where the $\mu$-th component of $\mathbf {X}_i$ equals the *i*-th component of ${\varvec{Q}}_\mu$ [30]. Similarly, the set $\{R_{\mu ,1}\}$ is described in terms of the *N*-dimensional vector $\mathbf {Y}$, where the $\mu$-th component of $\mathbf {Y}$ equals the $1^\text {st}$ component of ${\varvec{R}}_\mu$:

22 $$\begin{aligned} \{{\varvec{Q}}_\mu \}_{\mu =1,\ldots ,N} \quad\rightarrow & {} \quad \{\mathbf {X}_i\}_{i=1,\ldots ,D} \, , \end{aligned}$$

23 $$\begin{aligned} \{R_{\mu ,1}\}_{\mu =1,\ldots ,N} \quad\rightarrow & {} \quad \mathbf {Y}\, . \end{aligned}$$

With this, the instantaneous conformation tensor can be written as (see also [30])

24 $$\begin{aligned} \hat{c}_{ij} = \frac{1}{N} \mathbf {X}_i \cdot \mathbf {X}_j \, , \end{aligned}$$

and the partition function $\mathcal {G}$ becomes

25 $$\begin{aligned} \mathcal {G}= \int _{\varOmega _{Y}^{{\varvec{d}}}} \int _{\varOmega _{X}^{\mathbf {Y}}} \, \delta ^{(K)}\left( \hat{{\varvec{c}}}(\mathbf {X})-{\varvec{c}}\right) \, \prod _{\begin{array}{c} i=1 \\ \mu =1 \end{array}}^{D,N} dX_{i,\mu } \, \prod _{\mu =1}^N d Y_{\mu } \, ,\nonumber \\ \end{aligned}$$

with

26 $$\begin{aligned} \varOmega _{Y}^{{\varvec{d}}}= & {} \otimes ^{N} [d_1, d_2] \, , \end{aligned}$$

27 $$\begin{aligned} \varOmega _{X}^{\mathbf {Y}}= & {} \otimes ^{N} \left( [-2 Y_\mu , 2 Y_\mu ] \otimes {\mathbb R}^{(D-1)} \right) \, . \end{aligned}$$

We now introduce scaling factors $s_i$ ($i=1,\ldots ,D$) in the different spatial directions:

28 $$\begin{aligned} \mathbf {X}_i= & {} s_i \tilde{\mathbf {X}}_i \, , \qquad i=1,\ldots ,D \, , \end{aligned}$$

29 $$\begin{aligned} \mathbf {Y}= & {} s_1 \tilde{\mathbf {Y}}\, . \end{aligned}$$

Using $S \equiv \prod _{i=1}^D s_i$, and also with $\delta (a x) = (1/a) \delta (x)$ for $a > 0$, the partition function $\mathcal {G}$ can be written as (see [30] for the case without confinement)

30 $$\begin{aligned} \mathcal {G}= \mathcal {G}_1 \, \mathcal {G}_2 \, \mathcal {G}_3 \, , \end{aligned}$$

with

31 $$\begin{aligned} \mathcal {G}_1= & {} S^N \, s_1^N \, , \end{aligned}$$

32 $$\begin{aligned} \mathcal {G}_2= & {} \prod _{1 \le i \le j \le D} \frac{1}{s_i s_j} \nonumber \\= & {} \sqrt{ \left( \prod _{1 \le i, j \le D} \frac{1}{s_i s_j}\right) \left( \prod _{1 \le i \le D} \frac{1}{s_i^2}\right) } \nonumber \\= & {} \sqrt{ (S^2)^{-D} (S^2)^{-1} } \nonumber \\= & {} S^{(-D-1)} \, , \end{aligned}$$

33 $$\begin{aligned} \mathcal {G}_3= & {} \int _{\varOmega _{\tilde{Y}}^{{\varvec{d}}/s_1}} \int _{\varOmega _{\tilde{X}}^{\tilde{\mathbf {Y}}}} \, \delta ^{(K)}\left( \hat{{\varvec{c}}}(\tilde{\mathbf {X}})-\tilde{{\varvec{c}}}\right) \, \prod _{\begin{array}{c} i=1 \\ \mu =1 \end{array}}^{D,N} d\tilde{X}_{i,\mu } \, \prod _{\mu =1}^N d\tilde{Y}_{\mu } \, ,\nonumber \\ \end{aligned}$$

where $\mathcal {G}_1$ comes from the substitution of variables in the volume element, $\mathcal {G}_2$ originates from the substitution of variables in $\delta ^{(K)}$, and where we have defined $\tilde{c}_{ij} \equiv c_{ij}/(s_i s_j)$. This results in a scaling relation for $\mathcal {G}$,

34 $$\begin{aligned} \mathcal {G}({\varvec{c}},d_1,d_2) = S^{(N-D-1)} \, s_1^N \ \mathcal {G}(\tilde{{\varvec{c}}},d_1/s_1,d_2/s_1) \, . \nonumber \\ \end{aligned}$$

Based on Eq. (34), one obtains the scaling relation for the partition coefficient $\mathcal {W}$,

35 $$\begin{aligned} \mathcal {W}({\varvec{c}},d_1,d_2) = \mathcal {W}(\tilde{{\varvec{c}}},d_1/s_1,d_2/s_1) \, , \end{aligned}$$

where we have used the scaling behavior of the unconfined solution $\mathcal {G}_0$.

In the absence of confinement, the scaling argument is sufficient to determine $\mathcal {G}$ [30]. However, if confinement effects are included, the above scaling argument leads to a necessary rather than sufficient condition. It will be used in the numerical calculations presented further below, since it will allow us to eliminate one parameter from the simulations.

## The case of one dimension, $D=1$

In this section, we examine the behavior of the partition function $\mathcal {G}$ given in Eqs. (25)–(27) for $D=1$, and the consequences for the partition coefficient $\mathcal {W}$. In this case, we use the notation ${\varvec{c}}\rightarrow c$ and $s_1 \rightarrow s$. For $D=1$,

36 $$\begin{aligned} \hat{c}= \frac{1}{N} \mathbf {X}\cdot \mathbf {X}\, , \end{aligned}$$

and the partition function is given by

37 $$\begin{aligned} \mathcal {G}= \int _{\varOmega _{Y}} \int _{\varOmega _{X}} \, \delta \left( \hat{c}(\mathbf {X})-c\right) \, \prod _{\begin{array}{c} \mu =1 \end{array}}^{N} dX_{\mu } \, \prod _{\mu =1}^N d Y_{\mu } \, , \end{aligned}$$

with the integration domains given by the hyperrectangles

38 $$\begin{aligned} \varOmega _{Y}= & {} \otimes ^{N} [d_1, d_2] \, , \end{aligned}$$

39 $$\begin{aligned} \varOmega _{X}= & {} \otimes ^{N} [-2 Y_\mu , 2 Y_\mu ] \, . \end{aligned}$$

### Limiting cases

In qualitative terms, the dependence of the partition coefficient $\mathcal {W}$ on *c* can be rationalized as follows: For *c* smaller than a critical value $c_1$, one must have $\mathcal {W}= 1$ (i.e., $\mathcal {G}=\mathcal {G}_0$), since the extent of the dumbbells is too small to be able to feel the confinement yet. As *c* increases above $c_1$, $\mathcal {W}$ becomes smaller than unity; the more *c* increases, the more the value of $\mathcal {W}$ decreases, because the configurations of the dumbbells get increasingly susceptible to the confinement. This trend continues until *c* gets larger than a second critical value $c_2$, beyond which no configurations are permitted by the confinement, and thus $\mathcal {W}= 0$. These qualitative thoughts are formalized in the following.

For given value of the conformation “tensor” $\hat{c}$, the maximum extent of a dumbbell is in the situation that the value of $\hat{c}$ originates exclusively from a single dumbbell, say $\mu =1$, while all other dumbbells have zero extension; in this case, we have $\hat{c}= X_1^2/N$, i.e., $|X_1| = \sqrt{N \hat{c}}$. Therefore, the dumbbells cannot see the wall if $d_1> \sqrt{N \hat{c}} / 2$; one may thus write

40 $$\begin{aligned} \mathcal {W}= 1 \, , \quad \text {for} \ c < c_1\equiv 4 d_1^2 / N \, . \end{aligned}$$

**Fig. 3:**
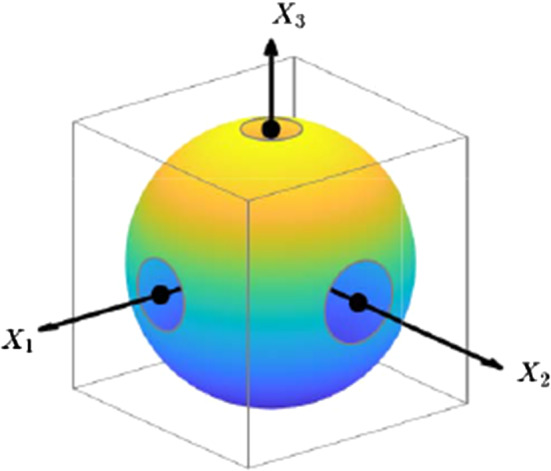
Limit 1: $c \searrow c_1$. Illustration of the situation for $N=3$; the spherical surface is defined by $c=\hat{c}$. The domain $\varOmega _{X}$ (box) is indicated by the gray edges

The onset of wall-effects can be quantified for cases where *c* is only slightly larger than $c_1$, i.e., when $\delta c \equiv c - c_1$ is small (see Fig. 3). As derived in Appendix A, the leading-order contribution in $\delta c > 0$ can be expressed as

41 $$\begin{aligned} \mathcal {W}\simeq & {} 1 - \gamma _N \frac{ 1 }{ d_1^{N} (d_2-d_1) } \left( \delta c \right) ^{(N+1)/2} \, , \end{aligned}$$

where $\gamma _N$ is a function of *N* only, see Eq. (A.18). The expression for $\mathcal {W}$ in Eq. (41) satisfies the scaling relation (35). It is noted that Eq. (41) applies only if $d_1> 0$; for $d_1\rightarrow 0$, the derivation presented in Appendix A does not hold. Instead, for $d_1\rightarrow 0$, we will closely examine the results of the numerical calculations for extracting the limiting behavior from there. In view of the exponent to $\delta c$ in Eq. (41), one can conclude that the sensitivity of the partition coefficient to confinement increases the larger the number of dumbbells.

For given $d_1$ and $d_2$, the largest dumbbell extension possible is $2 d_2$, which gives the contribution $4 d_2^2$ to $\hat{c}$. Therefore, no state in phase space exists with $\hat{c}> 4 d_2^2$, and the partition function must thus vanish,

42 $$\begin{aligned} \mathcal {W}= 0 \, , \quad \text {for} \ c > c_2\equiv 4 d_2^2 \, . \end{aligned}$$

**Fig. 4:**
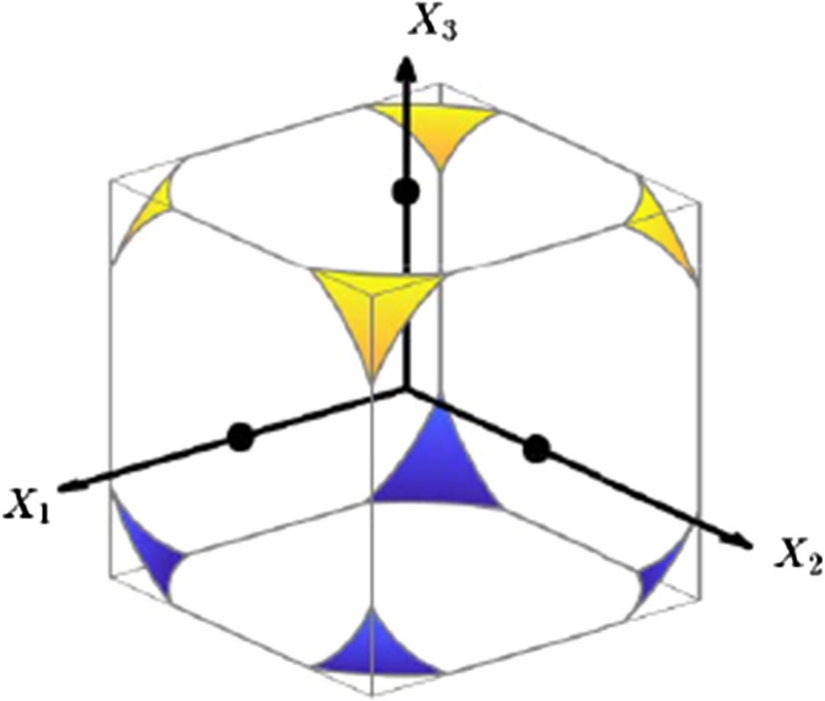
Limit 2: $c \nearrow c_2$. Illustration of the situation for $N=3$; the colored surfaces represent those parts of the (hyper-)sphere $c=\hat{c}$ which are contained in the domain $\varOmega _{X}$ (box indicated by the gray edges)

In order to study the behavior of $\mathcal {W}$ as *c* approaches $c_2$ from below (see Fig. 4), we introduce $\delta c = c-c_2$ (note: $\delta c$ is negative in the region of interest). According to Appendix B, the leading-order contribution in $\delta c < 0$ is given by

43 $$\begin{aligned} \mathcal {W}\simeq & {} \frac{N \check{\gamma }_N}{2^{N}} \frac{1}{d_2^{(3N-2)} (d_2-d_1)^{N}} (- \delta c)^{2N-1} \, , \end{aligned}$$

where $\check{\gamma }_N$ is a function of *N* only, given by Eq. (B.22). It is noted that this expression fulfills the scaling relation Eq. (35). The sensitivity of the partition coefficient to confinement in the limit $c \nearrow c_2$ increases rather strongly the larger the number of dumbbells, since the exponent to $\delta c$ in Eq. (43) is approximately four times as large as that in Eq. (41) for the case $c \searrow c_1$.

### Approximation of the partition coefficient $\mathcal {W}$

It is desirable to have a closed form expression for the partition coefficient $\mathcal {W}$ that represents the two analytically derived limits given in Eqs. (41) and  (43), and offers a relatively simple interpolation in between. To that end, it is useful to introduce the normalized value *u* of the conformation “tensor”,

44 $$\begin{aligned} u = \frac{c - c_1}{c_2- c_1} \, , \end{aligned}$$

where $0 \le u \le 1$ is the region in which the partition coefficient transitions from $\mathcal {W}=1$ to $\mathcal {W}=0$. The limiting behavior described by Eqs. (41) and  (43) of $\mathcal {W}$ at the boundaries of the interval [0, 1] can be expressed as

45 $$\begin{aligned} \mathcal {W}(u)\simeq & {} 1 - a \, u^{\alpha } \, , \qquad \ \ \, (u \searrow 0) \, , \end{aligned}$$

46 $$\begin{aligned} \mathcal {W}(u)\simeq & {} b \left( 1 - u\right) ^{\beta } \, , \qquad (u \nearrow 1) \, , \end{aligned}$$

for given values of the constants *a*, *b*, $\alpha$, and $\beta$,

47 $$\begin{aligned} a\equiv & {} {2^{(N+1)} \gamma _N} \frac{ \left( 1 - \frac{\lambda ^2}{N}\right) ^{(N+1)/2} }{ \lambda ^{N} (1-\lambda ) } > 0 \, , \end{aligned}$$

48 $$\begin{aligned} b\equiv & {} {2^{(3N-2)} N \check{\gamma }_N} \frac{\left( 1 - \frac{\lambda ^2}{N}\right) ^{(2N-1)}}{(1-\lambda )^{N}} > 0 \, , \end{aligned}$$

49 $$\begin{aligned} \alpha= & {} (N+1)/2 \ge 3/2 \, , \end{aligned}$$

50 $$\begin{aligned} \beta= & {} 2N-1 = 4 \alpha - 3 \ge 3 \, , \end{aligned}$$

where

51 $$\begin{aligned} \lambda \equiv \frac{d_1}{d_2}\, , \end{aligned}$$

with $$0 \le \lambda < 1$$.

In order to find a function that interpolates between these two limiting cases, we make two propositions:

- *Proposition 1:*
  52 $$\begin{aligned} \mathcal {W}_{\mathrm{ip},1}(u) = \left( \frac{ (1-u)^{\gamma _1} }{ (1-u)^{\gamma _1} + g_0 \left( 1-(1-u)^{\gamma _2}\right) ^{\gamma _3} }\right) ^{\gamma _4} \, .\nonumber \\ \end{aligned}$$
  If all parameters are positive, the limiting behavior of this function is given by
  53 $$\begin{aligned} \mathcal {W}_{\mathrm{ip},1}(u)\simeq & {} 1 - g_0 \gamma _2^{\gamma _3} \gamma _4 u^{\gamma _3} \, , \qquad (u \searrow 0) \nonumber \\ \end{aligned}$$
  54 $$\begin{aligned} \mathcal {W}_{\mathrm{ip},1}(u)\simeq & {} g_0^{-\gamma _4} (1-u)^{\gamma _1 \gamma _4} \, , \qquad \, (u \nearrow 1)\nonumber \\ \end{aligned}$$
  which can be used to find relations between the parameters $(a,b,\alpha ,\beta )$ and $(g_0,\gamma _1,\gamma _2,\gamma _3,\gamma _4)$.
- *Proposition 2:*
  55 $$\begin{aligned} \mathcal {W}_{\mathrm{ip},2}(u) = \left( \frac{ \left( 1-u^{\gamma _2}\right) ^{\gamma _1} }{ \left( 1-u^{\gamma _2}\right) ^{\gamma _1} + g_0 u^{\gamma _3} }\right) ^{\gamma _4} \, . \end{aligned}$$
  If all parameters are positive, the limiting behavior of this function is given by
  56 $$\begin{aligned} \mathcal {W}_{\mathrm{ip},2}(u)\simeq & {} 1 - g_0 \gamma _4 u^{\gamma _3} \, , \qquad \quad \ \ (u \searrow 0) \nonumber \\ \end{aligned}$$
  57 $$\begin{aligned} \mathcal {W}_{\mathrm{ip},2}(u)\simeq & {} g_0^{-\gamma _4} \gamma _2^{\gamma _1 \gamma _4} (1-u)^{\gamma _1 \gamma _4} \, , \quad (u \nearrow 1)\nonumber \\ \end{aligned}$$
  which can be used to find relations between the parameters $(a,b,\alpha ,\beta )$ and $(g_0,\gamma _1,\gamma _2,\gamma _3,\gamma _4)$.

It is noted that both propositions contain more parameters than needed for representing the two limits in Eqs. (45) and  (46), i.e., 5 instead of 4. However, this is done on purpose to have more flexibility in representing the numerical data also in intermediate regimes of *u* appropriately.

### Numerical calculations

For calculating the partition function $\mathcal {G}$, eqs. (37)–(39), which in turn determines the partition coefficient $\mathcal {W}$, we resort to numerical calculations. The idea is to sample the domain $\varOmega _{Y} \otimes \varOmega _{X}$ by placing points randomly (chosen from a homogeneous distribution in that domain), and making a histogram over the $\hat{c}$-values obtained.

Achieving high-fidelity results by sampling with randomly placed points is cumbersome in high dimensions, as demonstrated by the following two examples:

- Imagine a hypersphere with an inscribed (touching) hypercube. The fraction $\varphi$ of points in the hypersphere that are also inside of the hypercube decreases strongly with increasing space dimension *N*. For example, $\varphi (N=10) \simeq 4.0 \times 10^{-3}$ and $\varphi (N=100) \simeq 5.4 \times 10^{-31}$.
- Imagine a hypercube with an inscribed (touching) hypersphere. The fraction $\varphi$ of points in the hypercube that are also inside of the hypersphere decreases strongly with increasing space dimension *N*. For example, $\varphi (N=10) \simeq 2.5 \times 10^{-3}$ and $\varphi (N=100) \simeq 1.9 \times 10^{-70}$.

**Table 1:** Results of the numerical calculations for the behavior of the partition coefficient $\mathcal {W}$ in the limit described by Eq. (45), i.e., $u \searrow 0$ ($c \searrow c_1$)

	*N*	$\ln a$		$\alpha$		$R^2$	$u_{+}/u_{-}$
		theor.	sim.	theor.	sim.		
$\lambda = 0.8$	2	1.6599	$1.6399 \pm 0.0151$	1.5	$1.4985 \pm 0.0029$	0.9996	69.4079
	3	3.0154	$2.9036 \pm 0.0302$	2	$1.9860 \pm 0.0064$	0.9987	58.5570
	4	4.4517	$4.2729 \pm 0.0860$	2.5	$2.4804 \pm 0.0179$	0.9960	16.4446
	6	7.5903	$7.0606 \pm 0.2351$	3.5	$3.4297 \pm 0.0491$	0.9915	5.4739
	8	11.0247	$9.4886 \pm 0.5080$	4.5	$4.2550 \pm 0.1089$	0.9789	3.4212
	10	14.6939	$12.8510 \pm 0.8798$	5.5	$5.2420 \pm 0.1923$	0.9702	2.2255
$\lambda = 0$	2	–	$0.5854 \pm 0.0101$	–	$0.4998 \pm 0.0009$	0.9996	198.3434
	3	–	$0.9472 \pm 0.0103$	–	$0.4995 \pm 0.0009$	0.9996	336.9721
	4	–	$1.1964 \pm 0.0112$	–	$0.4982 \pm 0.0009$	0.9996	200.3368
	6	–	$1.5820 \pm 0.0121$	–	$0.4983 \pm 0.0009$	0.9996	112.1683
	8	–	$1.8749 \pm 0.0131$	–	$0.4995 \pm 0.0009$	0.9995	111.0522
	10	–	$2.0737 \pm 0.0138$	–	$0.4983 \pm 0.0010$	0.9995	90.9218
	20	–	$2.7705 \pm 0.0137$	–	$0.4995 \pm 0.0009$	0.9996	144.0269
	30	–	$3.1600 \pm 0.0155$	–	$0.4988 \pm 0.0009$	0.9995	91.8356
	40	–	$3.4281 \pm 0.0155$	–	$0.4977 \pm 0.0009$	0.9996	91.8356
	50	–	$3.6517 \pm 0.0164$	–	$0.4979 \pm 0.0009$	0.9995	91.8356
	60	–	$3.8387 \pm 0.0173$	–	$0.4983 \pm 0.0010$	0.9995	66.6863
	70	–	$3.9697 \pm 0.0167$	–	$0.4970 \pm 0.0009$	0.9996	78.2571
	80	–	$4.1167 \pm 0.0183$	–	$0.4978 \pm 0.0010$	0.9995	52.9845
	90	–	$4.2189 \pm 0.0173$	–	$0.4970 \pm 0.0009$	0.9996	47.9424
	100	–	$4.3030 \pm 0.0206$	–	$0.4960 \pm 0.0011$	0.9994	18.9158

For the parameters *a* and $\alpha$ in Eq. (45), simulation results (“sim.”) are compared with the theoretical predictions (“theor.”). Upper and lower bounds of the fitting range of *u* are denoted by $u_{+}$ and $u_{-}$

These examples emphasize that differences close to the domain boundary (e.g. corners) dominate the behavior at high dimensions. This will be relevant for the efficiency of the simulations discussed in the following.

In our simulations, MATLAB$^{\text{\textregistered }}$has been employed to calculate a histogram of *c* values, with bins that are equally spaced on the *c*-axis; apart from an overall (constant, i.e. *c*-independent) normalization, this histogram is equal to $\mathcal {G}$. In order to calculate $\mathcal {W}$, the histogram is normalized as follows: (i) Simulations for $c \searrow c_1$ and full range $c_1\le c \le c_2$: Simultaneously to the acquisition of the actual histogram, also a histogram is acquired as if the wall was absent, and so the ratio of these two histograms results in $\mathcal {W}$ (this procedure also ensures that $\mathcal {W}=1$ for $c < c_1$); (ii) Simulations for $c \nearrow c_2$: The histogram is divided by the (bin-average of the) analytically calculated $\mathcal {G}_0$ (note: there is an overall constant factor that cannot be calculated, and therefore the absolute magnitude of $\mathcal {W}$ cannot be determined in this case). The details of how the numerical calculations are performed can be found in Appendix D.

The scaling function $\mathcal {W}$ defined in Eq. (20) has four independent quantities in principle, namely ${\varvec{c}}$, $d_1$, $d_2$, and *N*. However, due to the scaling relation Eq. (35), it is sufficient to consider the case $s_1 = d_2$ and $s_i = 1$ for $i>1$, which leaves us with three independent quantities only, namely $\tilde{{\varvec{c}}} = {\varvec{c}}/d_2^2$, $\lambda$, and *N*. When presenting the numerical results further below, ${\varvec{c}}$ will be used in place of $\tilde{{\varvec{c}}}$, for simplicity.

The results of the numerical calculations for the limiting case $c \searrow c_1$ (i.e., $u \searrow 0$) are presented in Table 1, for the parameters *a* and $\alpha$ in Eq. (45). The simulations are set-up as to cover a range of *c*-values determined as follows, keeping in mind that $\mathcal {W}$ decreases from unity as *c* increases above $c_1$: The upper-bound for the *c*-range is chosen to ensure the $\mathcal {W}$-values sampled are in the interval [0.99, 1.00]; the lower bound for the *c*-range is chosen such that 90% of the sampled *c*-interval is above $c_1$, however, if that lower bound turns out to be negative it is reset to zero. In all simulations, the number of sampled *N*-dumbbell configurations is $n_\mathrm{cfg} = 10^{9}$, which are equally distributed among the bins of the histogram. The errors listed with the simulated data stand for the 95%-confidence interval. In order to judge the trustworthiness of the fits for extracting the parameters, the value of $R^2$ is tabulated, and also the ratio of the upper and lower bounds of the fitting range of *u* is specified, $u_{+}/u_{-}$. About the numerical results for the case $\lambda = 0.8$, the data in Table 1 show that the theoretical prediction and numerical results for the exponent $\alpha$ are in good agreement. The agreement for the prefactor *a* is less, and both for *a* and $\alpha$ it is noted that the agreement between prediction and simulation becomes less the higher the number of dumbbells *N*, which is due to limitations in the sampling efficiency the higher the dimension of the configuration space becomes. This decrease in accuracy is also reflected in the values for $R^2$ and $u_{+}/u_{-}$. For the case $\lambda = 0$, there is no theoretical prediction, however, the values $R^2$ and $u_{+}/u_{-}$ suggest that these results are a reasonable first attempt at discussing the behavior for $\lambda = 0$. Due to the lack of a theoretical prediction, simulations have been performed over a wide range of *N*. What stands out particularly from these results is that the exponent is quite close to $\alpha \simeq 0.50$.

The results for the limiting case $c \nearrow c_2$ (i.e., $u \nearrow 1$) are presented in Table 2, for the exponent $\beta$ in Eq. (46); the numerical procedure described in Appendix D for this limit does not allow to determine the prefactor *b* numerically. The simulations are set-up as to cover a range of *c*-values given by $[c_2(1+\epsilon ),c_2]$ with $\epsilon = -10^{-3}/N$. In all simulations, the number of sampled *N*-dumbbell configurations is $n_\mathrm{cfg} = 10^{9}$. The errors listed with the simulated data stand for the 95%-confidence interval. The trustworthiness of the results is again, as in Fig. 1, judged on the basis of $R^2$ and $u_{+}/u_{-}$. About the numerical results (which in this limit do not depend on $\lambda$), the data in Table 2 show that the theoretical prediction and numerical results for the exponent $\beta$ agree remarkably well for the values of *N* examined, and the values for $R^2$ and $u_{+}/u_{-}$ give confidence in these results. It should be mentioned that sampling at larger *N* becomes cumbersome due to increasing inefficiency of the sampling routine at higher dimensions.

**Fig. 5:**
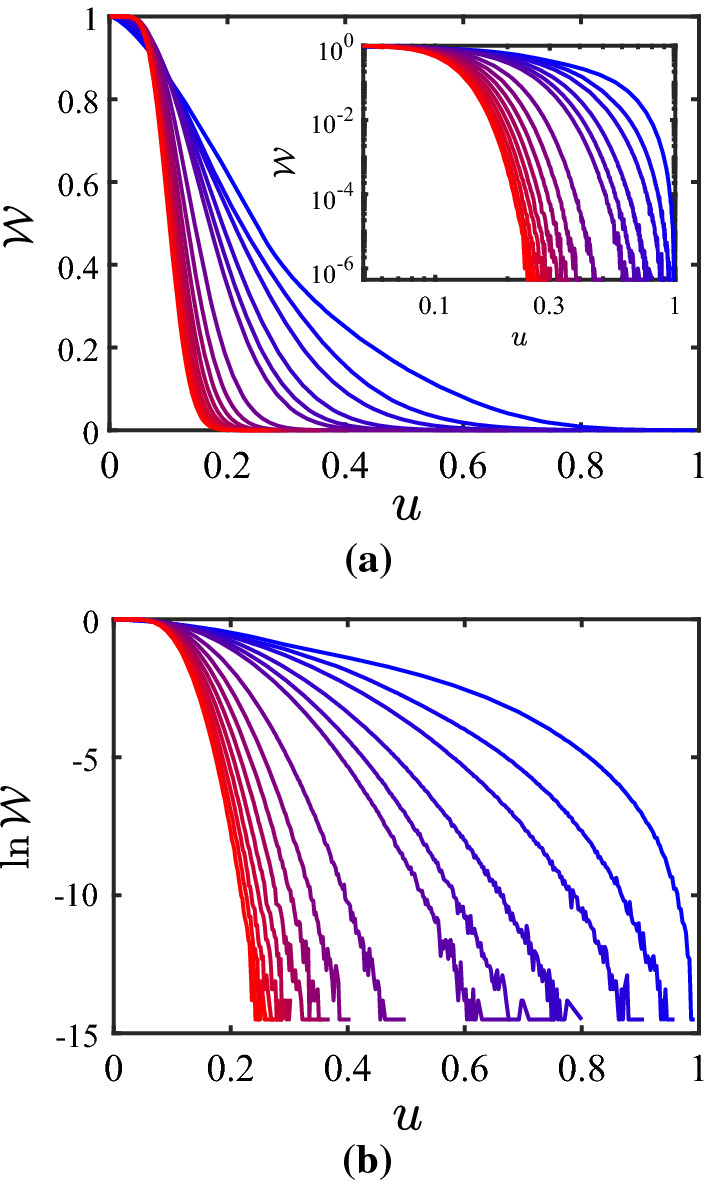
Partition coefficient $\mathcal {W}$ **a** and its logarithm **b** for $\lambda = 0.8$, and $N = 2$, 3, 4, 6, 8, 10, 20, 30, 40, 50, 60, 70, 80, 90, 100 (from blue to red, i.e., from right to left)

**Fig. 6:**
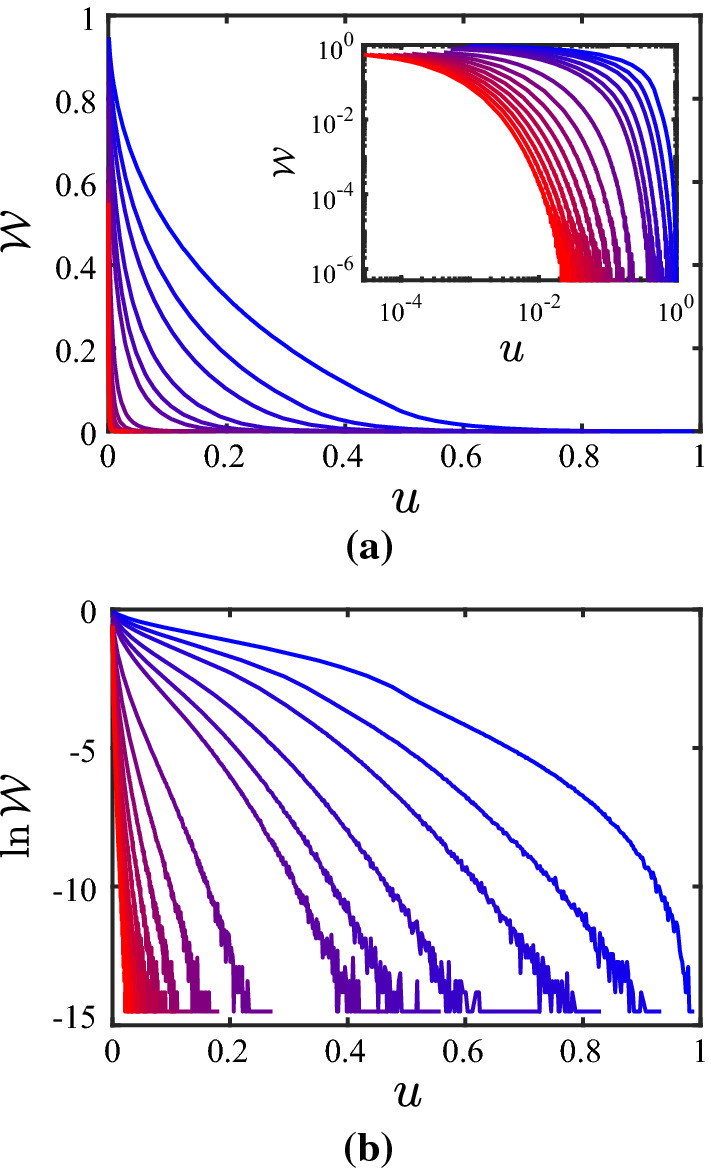
Partition coefficient $\mathcal {W}$ **a** and its logarithm **b** for $\lambda = 0$, and $N = 2$, 3, 4, 6, 8, 10, 20, 30, 40, 50, 60, 70, 80, 90, 100 (from blue to red, i.e., from right to left)

With the results presented in Tables 1 and  2 being in favorable agreement with the analytical predictions for the limiting cases, the next step consists in attempting to represent the partition coefficient $\mathcal {W}$ over the entire range with the proposed functions given by Eqs. (52) and  (55). Figures 5 and  6 show the results for the behavior of $\mathcal {W}$ versus *u* for $\lambda = 0.8$ and $\lambda = 0$, respectively, for various values of *N*; subfigures (b) are shown as they directly illustrate the change in the configurational entropy $\varDelta S_\mathrm{c}$, see Eq. (21). The data shown in Figs. 5 and  6 is available via a repository [31]. With respect to fitting the behavior of $\mathcal {W}$ with the functions given by Eqs. (52) and  (55), the following conclusions can be drawn from several of such attempts: When constraining the parameter set in the fit functions by the behavior in the two limiting cases, both of the fit functions give a poor representation of the overall behavior. By “poor” we mean that the error defined by $\epsilon _\mathcal {W}= \Vert \mathcal {W}- \mathcal {W}_\mathrm{fit} \Vert / \Vert \mathcal {W}\Vert$ with $\Vert \ldots \Vert$ the $L^2$-norm over the *u*-interval [0, 1] is as large as 5% until 50%. This holds not only when setting $\gamma _4 = 1$, but even when $\gamma _4$ is included as an additional degree of freedom in the fitting procedure. This being said, one can also try to fit the numerical data for $\mathcal {W}(u)$ with the functions given by Eqs. (52) and  (55) without enforcing the two limiting cases; therefore, in this case, the free parameters are $g_0$, $\gamma _1$, $\gamma _2$, and $\gamma _3$, while we skip $\gamma _4$ by setting $\gamma _4=1$. The results of these fits are represented in Table 3. The optimization of the parameters is done with the Global Optimization Toolbox in Matlab^®^, particularly using GlobalSearch. Beyond the value of $R^2$, the quality of the fit is judged also in terms of the following two quantities: For each combination of $\lambda$, *N* and $\mathcal {W}_{\mathrm{ip},\#}$, the GlobalSearch is run five times, where each GlobalSeach run performs a large number of solver runs (see Matlab^®^ manual for details). If not 100% of all solver runs converged successfully, the criterion “conv.” in the table is specified as “n” (for “no”), and by “y” (for “yes”) otherwise. Furthermore, for the cases where not all five GlobalSearch runs resulted in the same solution, another five runs have been performed; the number of different (but converged) solutions from these ten runs is listed in the column “# sol.” (for “number of solutions”). If both all solver searches converged and only one solution was found, the corresponding entry in the column “conv./# sol.” is left empty. Looking at the values for $R^2$ in Table 3, it appears that the fits are rather successful. However, this should be taken with caution, for two reasons: First, the columns for “conv./# sol.” indicate problems in the convergence. And second, there is non-monotonous behavior in the *N*-dependence of the parameters, except for $\mathcal {W}_{\mathrm{ip},1}$ at $\lambda = 0.8$. In several of these cases, it seems that there are different branches for solutions, which are almost equally good. However, since it is not clear a priori which branch is the good one, one should refrain from directing the numerical-solution finding in a particular direction. Furthermore, it may also be the case that the functional forms of the fit-functions are not suitable. Since we cannot represent the limiting cases (Eqs. (45) and  (46)) anyway, one should view the results presented in Table 3 just as a way of representing the data (e.g., the reader can take the parameters, and “reconstruct” the data).

**Table 2:** Results of the numerical calculations for the behavior of the partition coefficient $\mathcal {W}$ in the limit described by Eq. (46), i.e., $u \nearrow 1$ ($c \nearrow c_2$)

*N*	$\beta$		$R^2$	$u_{+}/u_{-}$
	theor.	sim.		
2	3	$2.9993 \pm 0.0002$	1.0000	198.3434
3	5	$4.9974 \pm 0.0030$	1.0000	37.3376
4	7	$6.9994 \pm 0.0058$	1.0000	17.8143
6	11	$10.9991 \pm 0.0068$	1.0000	7.0993
8	15	$15.0056 \pm 0.0116$	0.9999	5.9895
10	19	$19.0069 \pm 0.0155$	0.9999	3.8190

For the parameter $\beta$ in Eq. (46), simulation results (“sim.”) are compared with the theoretical prediction (“theor.”). Upper and lower bounds of the fitting range of *u* are denoted by $u_{+}$ and $u_{-}$

**Table 3:** Results for the fit parameters in the functions Eqs. (52) and  (55), for $\gamma _4=1$, representing the partition coefficient $\mathcal {W}$ over the entire range

	*N*	$\mathcal {W}_{\mathrm{ip},1}$						$\mathcal {W}_{\mathrm{ip},2}$					
		$g_0$	$\gamma _1$	$\gamma _2$	$\gamma _3$	$R^2$	$\text {conv./}\,$	$g_0$	$\gamma _1$	$\gamma _2$	$\gamma _3$	$R^2$	$\text {conv./}\,$
							# $\text {sol.}$						# $\text {sol.}$
$\lambda = 0.8$	2	1.1928	2.6233	3.0801	1.7401	0.9997		11.474	2.4738	2.1898	1.8424	0.9996	
	3	0.29940	5.7119	9.0985	2.3215	1.0000		18.731	9.0034	2.9765	2.0277	1.0000	
	4	0.19580	7.6702	11.709	2.6886	1.0000		25.498	12.821	2.8020	2.1761	1.0000	
	6	0.12040	10.657	15.222	3.4780	1.0000		56.798	24.930	3.0069	2.5400	1.0000	
	8	0.085000	13.128	18.629	4.4765	1.0000		117.98	46.735	3.3285	2.8518	1.0000	
	10	0.064600	15.283	21.738	5.5539	1.0000		213.82	79.445	3.5845	3.0948	1.0000	
	20	0.027563	23.387	33.946	11.692	1.0000	n/1	1539.6	564.05	4.4486	3.8405	1.0000	
	30	0.016810	29.217	42.706	18.390	1.0000		5202.5	2069.0	4.9449	4.2612	1.0000	
	40	0.011761	33.934	50.118	26.088	1.0000	n/1	6373.8	859.02	4.0251	4.2792	1.0000	y/3
	50	0.0089302	37.912	56.624	34.988	1.0000		9997.9	976.31	3.9146	4.4084	1.0000	
	60	0.0071426	41.378	61.928	43.429	1.0000		9999.9	569.41	3.4151	4.3795	0.9999	
	70	0.0058933	44.481	67.436	54.448	1.0000		72435	38122	5.8897	5.0895	1.0000	
	80	0.0049848	47.307	72.445	66.697	1.0000		16520	665.98	3.3110	4.5132	0.9999	
	90	0.0043054	49.888	76.870	78.996	1.0000		21793	774.93	3.3276	4.5917	0.9999	
	100	0.0037888	52.245	81.034	92.281	1.0000	n/1	30666	1023.2	3.4289	4.6919	0.9999	n/1
$\lambda = 0$	2	0.79305	4.5358	11.462	0.63303	0.9998	n/1	4.4637	5.6696	1.8048	0.67676	0.9998	
	3	0.98629	6.9145	15.313	0.61643	0.9998		6.4233	8.5624	1.5821	0.65565	0.9998	
	4	1.2496	8.9914	16.461	0.60102	0.9999		8.0619	10.124	1.3873	0.63421	0.9998	
	6	2.1212	12.407	13.097	0.57660	0.9998		9.2815	10.741	1.0551	0.57359	0.9998	
	8	10.590	13.756	1.3409	0.57019	0.9997	n/1	8.4841	10.627	0.83214	0.50899	0.9997	
	10	20.505	17.931	0.69208	0.57350	0.9995	n/1	6.6469	10.630	0.68290	0.44177	0.9998	
	20	31.500	53.280	0.82174	0.51876	0.9987	n/1	0.82575	13.524	0.40339	0.12526	0.9999	
	30	11.092	108.42	13.901	0.50955	0.9986	y/5	0.74724	18.893	0.40177	0.093411	1.0000	
	40	12.073	178.57	21.810	0.50979	0.9985	y/5	0.96736	24.689	0.41607	0.10642	1.0000	
	50	12.649	264.31	32.082	0.50688	0.9986		1.4234	31.908	0.44050	0.12839	1.0000	
	60	13.113	369.53	43.418	0.50550	0.9985		1.0929	35.855	0.42533	0.10012	1.0000	
	70	13.338	489.44	58.914	0.50544	0.9986		1.2813	42.051	0.43375	0.10754	1.0000	
	80	13.368	628.26	78.658	0.50853	0.9986		155.41	1075.1	1.1037	0.53137	0.9981	
	90	13.650	788.19	96.276	0.51065	0.9985		174.93	1327.0	1.0964	0.53147	0.9980	
	100	13.834	962.67	117.25	0.51156	0.9985		195.73	1610.5	1.0920	0.53141	0.9980	y/3

Symbols are explained in the text

## The case of higher dimensions, $D > 1$

The effect of confinement on the configuration of *N* dumbbells in higher dimensions, $D>1$, is closely related to the case $D=1$ discussed above, as shown in the following. Consider the expression $\hat{c}_{ij} = \mathbf {X}_i \cdot \mathbf {X}_j / N$, introduced in Eq. (24). The effect of $\mathbf {Y}$ does not need to be considered in the following, except for the fact that it restricts the domain of $\mathbf {X}_1$. In the high-dimensional space with vectors $(\mathbf {X}_1, \ldots , \mathbf {X}_D)$, the quantities $\hat{c}_{ij}$ measure the lengths of and angles between the *X*-vectors for $i=j$ and $i \ne j$, respectively. The conditions $\hat{c}_{ij} = c_{ij}$ are therefore rotationally invariant in this high-dimensional space. In contrast, the presence of the confining wall restricts the vector $\mathbf {X}_1$, as discussed in detail in the previous Sect. 4: While all vectors $\mathbf {X}_1$ with $\hat{c}_{11} \le c_1$ are admissible, all $\mathbf {X}_1$ with $\hat{c}_{11} > c_2$ are impossible; in between, it depends on the orientation of $\mathbf {X}_1$, i.e., on the components of $\mathbf {X}_1$ for given length of the vector, whether it is admissible–the remainder of this argument will thus focus on this intermediate range, $c_1 < \hat{c}_{11} \le c_2$. Since the wall does not put any constraints on $\mathbf {X}_i$ for $i > 1$, the number of states satisfying $\hat{c}_{ij} = c_{ij}$ is reduced by the wall only because of its restrictions on the orientation of $\mathbf {X}_1$. Therefore, once the reduction of states due to the restrictions on the orientation of $\mathbf {X}_1$ are taken into account, there is no further wall effect on the partition function $\mathcal {G}$. In other words: For any arbitrary set $(\mathbf {X}_1, \ldots , \mathbf {X}_D)$ which is compatible with all conditions $\hat{c}_{ij} = c_{ij}$, one can impose an overall rotation (which will not affect the $\hat{c}_{ij} = c_{ij}$) in such a way as to ensure that $\mathbf {X}_1$ is admissible, i.e., that also the wall condition is respected; the fact that for some $\mathbf {X}_1$ this “corrective” rotation is needed is indicative of the reduction of states due to the wall, i.e., representative of $\mathcal {W}$ being smaller than unity. Therefore, the prediction is that

58 $$\begin{aligned} \mathcal {G}({\varvec{c}}) = \mathcal {W}(c_{11}) \, \mathcal {G}_0({\varvec{c}}) \, , \qquad \text {for} \ D \ge 1, \end{aligned}$$

where $\mathcal {W}$ denotes the partition coefficient discussed for $D=1$ in Sect. 4. For verifying this prediction in the numerical calculations, one can select any value for $c_{11}$ with a corresponding value $\mathcal {W}(c_{11})$ in the interval $]c_1,c_2]$, and plot the quantity $\mathcal {G}({\varvec{c}}) / \mathcal {G}_0({\varvec{c}})$ as a function of the other components of ${\varvec{c}}$; the result should be constant and give the value $\mathcal {W}(c_{11})$.

The numerical calculations for the example case $D=2$ have been performed as follows: (i) Select the value for $c_{11}$ such that, according to the 1D calculations, one obtains $\mathcal {W}= 0.1$, $\mathcal {W}= 0.5$, and $\mathcal {W}= 0.9$, respectively (these values are just taken as typical examples, for the purpose of illustration); these values are called $\mathcal {W}_\mathrm{target}$ and are listed, together with the corresponding value for $c_{11}$, in Table 4. (ii) For each of the $n_\mathrm{cfg} = 10^9$ configurations generated, proceed as follows: Choose $\mathbf {X}_1$ from a homogeneous distribution on the surface of the hypersphere with radius $\sqrt{N c_{11}}$; choose $\mathbf {X}_2$ from a homogeneous distribution in the hypersphere with radius $\sqrt{N c_{22, \mathrm{max}}}$ – here, we use $c_{22, \mathrm{max}} = c_2= 4 d_2^2$; given these $\mathbf {X}_1$ and $\mathbf {X}_2$, calculate the corresponding values of $c_{12}$ and $c_{22}$, and correspondingly update the histogram for $\mathcal {G}_0$ in which the wall effect is neglected; for $\mathbf {Y}$ drawn from a homogeneous distribution on $\varOmega _Y$, check if $|X_\mu | \le 2 Y_\mu$ and, if this condition is fulfilled, also update the histogram for $\mathcal {G}$ in which the wall effect is included.

**Table 4:** Quantities in relation to the numerical verification of Eq. (58) for $D=2$ and $N=10$, and shown in Figs. (7) and  (8)

	$c_{11}$	$\mathcal {W}_\mathrm{target}$	$\langle \mathcal {G}/\mathcal {G}_0 \rangle$	$\sigma _{\mathcal {G}/\mathcal {G}_0}$
$\lambda = 0.8$	0.5800	0.9000	0.8998	0.0017
	0.8895	0.5000	0.5002	0.0024
	1.2920	0.1000	0.1000	0.0011
$\lambda = 0$	0.000652	0.9000	0.9001	0.0012
	0.025956	0.5000	0.5000	0.0026
	0.223790	0.1000	0.1001	0.0017

**Fig. 7:**
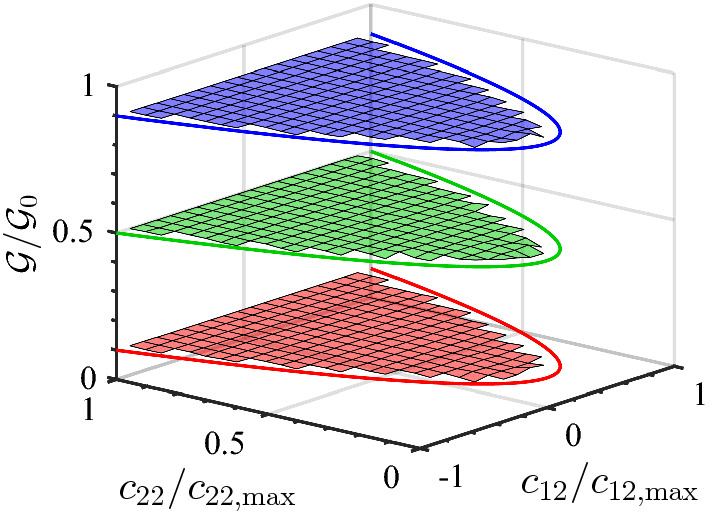
Numerical results for the ratio $\mathcal {G}/\mathcal {G}_0$ for $D=2$, $N=10$, and $\lambda = 0.8$, for three values of $c_{11}$ listed in Table 4

**Fig. 8:**
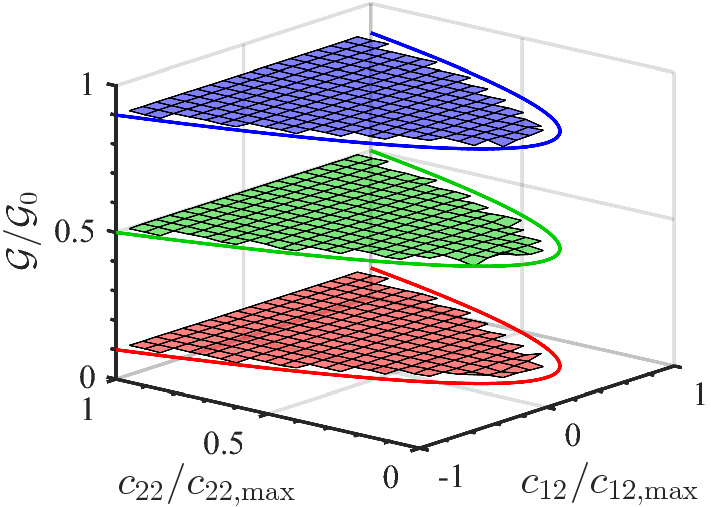
Numerical results for the ratio $\mathcal {G}/\mathcal {G}_0$ for $D=2$, $N=10$, and $\lambda = 0$, for three values of $c_{11}$ listed in Table 4

The average $\langle \mathcal {G}/\mathcal {G}_0 \rangle$ and standard deviation $\sigma _{\mathcal {G}/\mathcal {G}_0}$ are calculated over the bins shown in Figs. 7 and  8, respectively

Figures 7 and  8 show the results of the numerical calculations for $D=2$ and $N=10$. Based on the relation between the components of $\hat{{\varvec{c}}}$ and the vectors $\mathbf {X}_1$ and $\mathbf {X}_2$, it is obvious that $\det {\varvec{c}}\ge 0$, i.e., $c_{11} c_{22} \ge c_{12}^2$. Particularly, since $c_{11}$ is fixed, the boundary of this domain can be expressed in the form $(c_{22}/c_{22, \mathrm{max}}) = \left( c_{12}/c_{12, \mathrm{max}}\right) ^2$ with $c_{22, \mathrm{max}}$ defined above and $c_{12, \mathrm{max}} = \sqrt{c_{11} c_{22, \mathrm{max}}}$–these are the solid lines in Figs. 7 and  8. As this boundary is approached from within the admissible ${\varvec{c}}$-range, the number of admissible states decreases drastically, and therefore the region close to the boundary has decreased accuracy due to limited sampling; in the figures, we therefore include only those bins which are contained entirely in the admissible domain.

The results in Fig. 7 for $\lambda =0.8$ and in Fig. 8 for $\lambda =0$ show that for given $c_{11}$ the ratio $\mathcal {G}/\mathcal {G}_0$ indeed is independent of the other components, $c_{12}$ and $c_{22}$, as expected. Furthermore, the (constant) value of the ratio $\mathcal {G}/\mathcal {G}_0$ agrees with the value of $\mathcal {W}$ (from 1D) for the corresponding value of $c_{11}$, again as expected: As the quantitative results in Table 4 show, the expected $\mathcal {W}$-values are met within the standard deviation $\sigma _{\mathcal {G}/\mathcal {G}_0}$, and the smallness of the standard deviation justifies calling the ratio $\mathcal {G}/\mathcal {G}_0$ being independent of $c_{12}$ and $c_{22}$. The finding that the ratio $\mathcal {G}/\mathcal {G}_0$ depends only on the component of ${\varvec{c}}$ in the direction normal to the wall is in agreement with earlier results by Mavrantzas and Beris [8, 10].

## Discussion and outlook

In this paper, the effect of confinement on the conformation of *N* non-interacting dumbbells in *D* dimensions close to a non-interacting and rigid wall has been examined. To that end, the partition function $\mathcal {G}$, partition coefficient $\mathcal {W}$, and the confinement-induced change in the configurational entropy $\varDelta S_\mathrm{c}$ have been studied as a function of the conformation tensor ${\varvec{c}}$ of the dumbbells. In the case $D=1$ (Sect. 4), analytical predictions have been derived for $\mathcal {W}$ in two limiting cases ($c \searrow c_1$ and $c \nearrow c_2$), and the numerical calculations have been found to be in favorable agreement with the predictions, particularly as far as the exponents in the scaling relations are concerned. For the case where an analytical prediction has not been achieved ($\lambda =0$, $c \searrow c_1$), extensive numerical calculations give trustworthy results for this limiting case for a wide range of *N*. Beyond these limiting cases, the overall behavior of the partition coefficient $\mathcal {W}$ has been examined as well. In this case, it has been found that the fit-functions proposed with a minimal set of parameters give an unsatisfactory representation of the results from the numerical calculations. Concretely, a compact analytic expression with a small number of parameters that represents both the limiting cases as well as the overall behavior is still not found, but this is definitely worth pursuing in future studies. Furthermore, it has been shown in Sect. 5 that the effect of confinement for $D>1$ is captured completely by the partition coefficient $\mathcal {W}$ determined for $D=1$; this has been proven analytically, as well as demonstrated on the basis of numerical calculations for $D=2$.

**Fig. 9:**
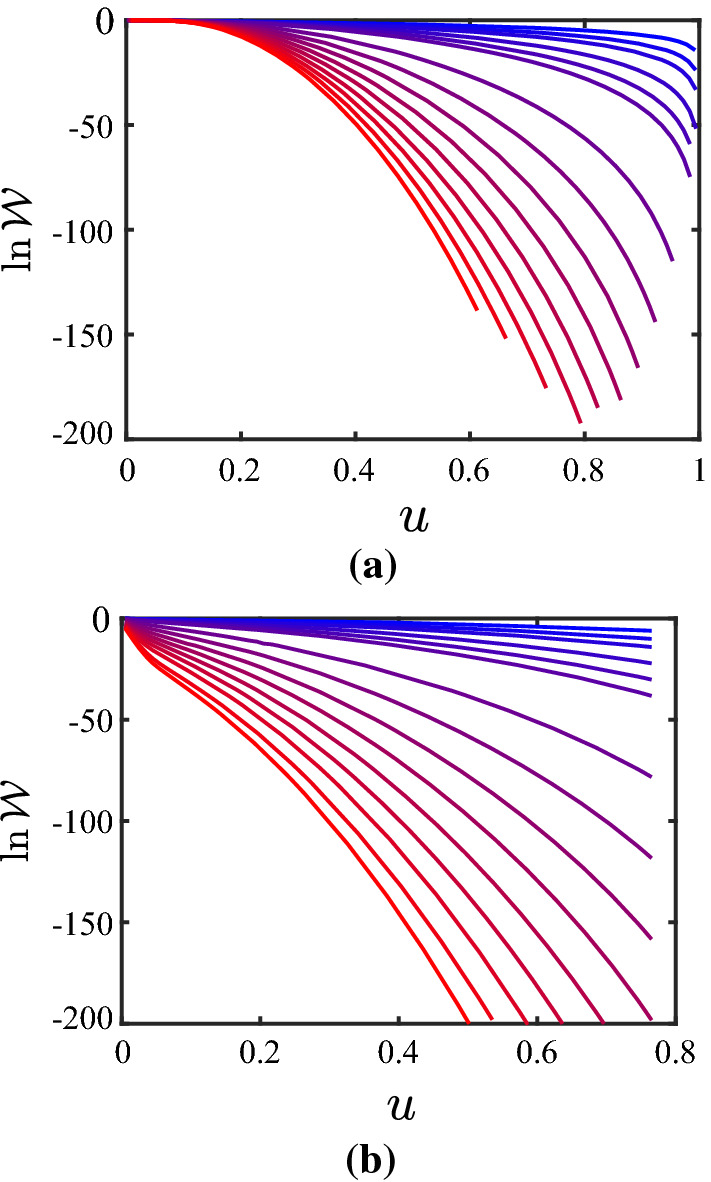
Logarithm of the partition coefficient, $\ln \mathcal {W}$, as obtained by combining the data in Figs. 5 and  6, respectively, with the results from the Wang-Landau simulations, **a** for $\lambda = 0.8$ and **b** for $\lambda = 0$, and $N = 2$, 3, 4, 6, 8, 10, 20, 30, 40, 50, 60, 70, 80, 90, 100 (from blue to red, i.e., from upper-right to lower-left)

Inspection of Figs. 5 and 6 shows clearly that the simulation strategy proposed in the main part of this paper—while following closely the philosophy of the analytical calculations—has its limitations: Only limited ranges of (rescaled) conformation *u* and $\ln \mathcal {W}$ (which relates to the wall-induced change of the entropy, see Eq. (21)) can be captured adequately, the situation becoming more severe the higher the number *N* of dumbbells. In order to extend the simulations to a wider range of conformations, one can use techniques that counteract systematically the sampling inefficiency in remote and deserted parts of phase space. In order to point out a possible route towards improvement, it is chosen here to use the algorithm of Wang and Landau [32, 33], with the improved $t^{-1}$ algorithm to avoid saturation [34, 35], as an illustrative example. While we refer the reader to these original publications for details about the technique in general, here only the specifics for its application in this outlook are mentioned: 100 equally spaced bins are considered in the *c*-range $[0,c_2]$; proposing a new state consists of choosing one of the 2*N* beads at random and perturbing its position with an increment from a homogeneous distribution on $[-0.2,0.2]$; the histogram *H* is checked every 1000 Monte-Carlo steps for flatness, the latter being taken as $\mathrm{min}(H) \ge 0.8 \, \mathrm{max}(H)$; and the final accuracy of the results is set by $\mathrm{ln}f_\mathrm{final} = 10^{-7}$. The simulations are used to calculate the quantity $\mathcal {G}$, which in turn is divided by the analytically calculated $\mathcal {G}_0$ to obtain the partition coefficient $\mathcal {W}$; however, so doing $\mathcal {W}$ is known only up to a multiplicative factor, an issue that is inherent to the Wang-Landau procedure; in turn, $\ln \mathcal {W}$ is known only up to an additive constant. As a remedy, it is noticed that the procedure presented earlier in this paper does not suffer from this shortcoming, and therefore the overlap-region in $\ln \mathcal {W}(u)$ of these two procedures can be used to shift the Wang-Landau results appropriately. In this illustrative example, only in a few cases ($\lambda =0$: $N=70$, $N=80$, $N=90$, $N=100$), there is no overlap but rather a gap between the two sets of data (the lower end of the range covered in the Wang-Landau simulations being $u=0.04$ for $N=70$, and $u = 0.05$ for $N=80$, $N=90$, $N=100$); therefore a quadratic function is used to interpolate between the two datasets, matching the slopes on both sides of the gap, as well as the (known) absolute value of $\ln \mathcal {W}$ adjacent to the left end of the gap, for the sake of illustration. The behavior of $\ln \mathcal {W}$ obtained by shifting the Wang-Landau results and merging them with our previously obtained results are shown in Fig. 9; the corresponding data is available via a repository [31]. The substantial increase in range covered is apparent when comparing these results with the ones presented in Figs. 5b and  6b, respectively. In summary, it is a promising route forward to combine the simulations presented in the main part of this paper with, e.g., Wang-Landau sampling in order to obtain the function $\ln \mathcal {W}(u)$ at high fidelity over a wide range of the dumbbell conformation, particularly for larger numbers *N* of dumbbells.

The configurational entropy and free energy play a key role in the modeling of the dynamics of complex fluids, as explained in the Introduction Sect. 1; e.g., the derivative of these quantities with respect to the conformation tensor act as driving forces for structural relaxation. In general, the partition coefficient $\mathcal {W}$ is constant (unity) for small dumbbell extensions, and thereafter decreases the larger the dumbbell extension perpendicular to the wall (e.g., see Figs. 5 and  6). This means that, in this second range, the configurational entropy (see also Eq. (21)) is lowered by the confinement, and it is lowered more the larger the dumbbell extension; in turn, the free energy is increased by the confinement, the increase being stronger the larger the dumbbell extension. This implies that the extension of the dumbbells in the direction perpendicular to the wall is compressed as a result of the confinement. The closer the center-of-mass of the dumbbell to the wall, the stronger the confinement–in full agreement with Mavrantzas and Beris [8, 10]–, and in the limit of the dumbbells being basically at the wall one finds that the dumbbell extension in the perpendicular direction vanishes, in agreement with the results presented in figure 2in [10]. For a more quantitative comparison with their results, slabs of equal thickness at different distances from the wall would have to be studied, followed by the calculation of the average conformation tensor in each slab; in contrast, in our study, we have restricted our attention to results for a fixed ratio $\lambda =d_1/d_2$, for the purpose of illustration ($\lambda =0$ and $\lambda =0.8$). The effect of confinement on the dumbbell conformations can also be illustrated by calculating the confinement contribution to the driving forces for structural relaxation, here written for $D=1$,

59 $$\begin{aligned} \frac{\partial (\varDelta S_\mathrm{c})}{\partial c} \propto \left\{ \begin{array}{ll} - (c-c_1)^{(N-1)/2} \, , &{} \ \text {for} \quad c \searrow c_1, 0< d_1< d_2\, , \\ - c^{(\alpha _\mathrm{sim}-1)} \, , &{} \ \text {for} \quad c \searrow 0, \ 0 = d_1< d_2\, , \\ -1/(c_2-c) \, , &{} \ \text {for} \quad c \nearrow c_2, 0 \le d_1< d_2\, , \end{array}\right. \nonumber \\ \end{aligned}$$

based on Eqs. (41),  (43), and the numerical results presented in Table 1 from which one infers the exponent $\alpha _\mathrm{sim} = 0.50$. About these expressions, it is to be noted that only in the first of the three cases, the driving force does not diverge in the limit, the strongest divergence being as *c* approaches the upper limit$c_2$, beyond which no configurations are possible anymore. Furthermore, only in the first case, the exponent of the limiting behavior depends on the number of dumbbells *N*.

The analysis in this paper has been performed for a finite number *N* of dumbbells. The following prediction can be formulated for the thermodynamic limit, $N \rightarrow \infty$: As shown in Figs. 5 and  6, the transition from no confinement ($\mathcal {W}=1$) to full confinement ($\mathcal {W}=0$) occurs in a narrower region the higher the value of *N*. This conclusion is also supported by the predictions in Eqs. (41),  (43) for the limiting behavior. It is pointed out that the non-trivial *N*-dependence of the partition coefficient $\mathcal {W}$ originates from the mutual coupling of the dumbbells due to the constraint $\hat{{\varvec{c}}}= {\varvec{c}}$ when counting the microstates in the statistical-mechanics calculation, because the instantaneous conformation tensor $\hat{{\varvec{c}}}$ depends on all dumbbells simultaneously (see Eq. (3)).

All of the above is valid if the confinement is given by a single flat wall. However, let us imagine that there is a second flat wall, parallel to the first one and located at $d_3$, i.e., the dumbbells are confined to a slab of width $d_3$. Our calculations have shown that conformations with $c > c_2$ are not admissible, which amounts to $\mathcal {W}=0$. The extreme case $c = c_2$ corresponds to the situation where all dumbbells have their center-of-mass at $d_2$ from the wall and each dumbbell has the maximum extension $2d_2$. However, this implies that the beads (i.e., ends) of the dumbbells can reach the second wall only if $d_3 < 2d_2$. A ramification of this finding is that, when performing conformation-tensor based viscoelastic flow calculations in a narrow slab, one must be careful that no volume element crosses the center-plane between the two confining walls; if this condition is respected, the results of this paper can be used readily for slab-confinements as well.

Not only are the dumbbells compressed in the direction normal to the wall the closer they are located to the wall, see above discussion; in addition, dumbbell depletion occurs as well. Given the Helmholtz free energy in terms of the conformation tensor ${\varvec{c}}$ and the number (density) of dumbbells, the simultaneous occurrence of dumbbell compression and depletion can be addressed, e.g. at equilibrium, by minimizing the free energy with respect to ${\varvec{c}}$ and at the same time keeping the chemical potential of the dumbbells constant, see [8, 10] for details. While in this paper it has been chosen to perform the statistical-mechanics calculation for a constant number of dumbbells, an alternative direction for future work is to consider the case of a fixed chemical potential instead.

## Figures and Tables

**Fig. 10:**
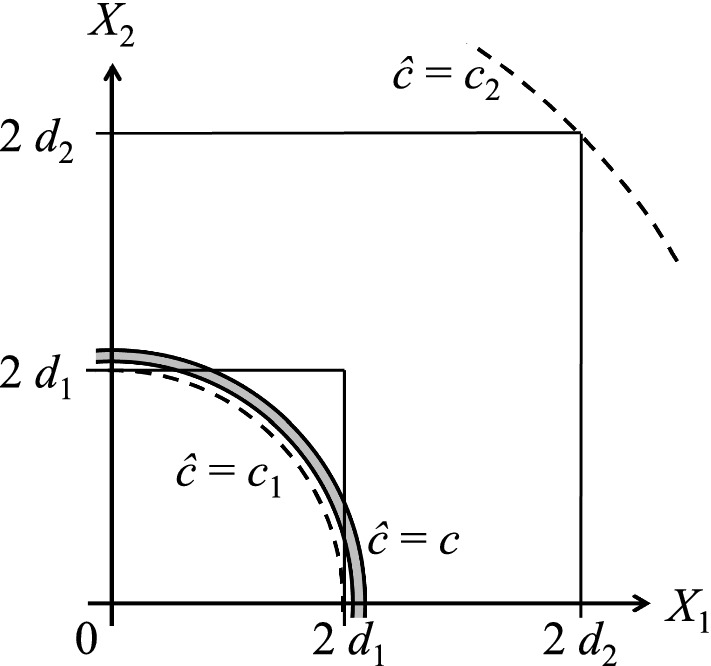
Illustration of the situation examined in Appendix A, for $N=2$; the spherical surface is defined by $\hat{c}=c$

**Fig. 11:**
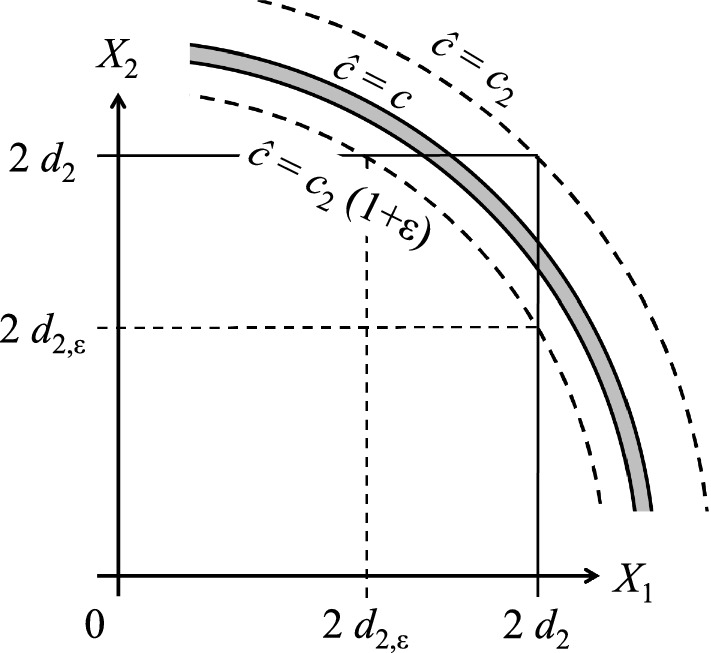
Illustration of the situation examined in Appendix B, for $N=2$; the spherical surface is defined by $\hat{c}= c$

## Partition function: Limit 1

In this appendix, the partition function $\mathcal {G}$ is examined for $D=1$ in the limit $c \searrow c_1$ with $c_1= 4 d_1^2 / N$. We start by writing

A.1 $$\begin{aligned} \mathcal {G}= \int _{\varOmega _{Y}} \mathcal {G}_{\mathbf {Y}} \, \prod _{\mu =1}^N d Y_{\mu } \, , \end{aligned}$$

with

A.2 $$\begin{aligned} \mathcal {G}_{\mathbf {Y}} = \int _{\varOmega _{X}} \, \delta \left( \hat{c}(\mathbf {X})-c\right) \, \prod _{\begin{array}{c} \mu =1 \end{array}}^{N} dX_{\mu } \, . \end{aligned}$$

and

$$\begin{aligned} \varOmega _{Y}= & {} \otimes ^{N} [d_1, d_2] \, , \\ \varOmega _{X}= & {} \otimes ^{N} [-2 Y_\mu , 2 Y_\mu ] \, . \end{aligned}$$

As explained in detail in [30], the expression for the partition function consists of a surface area in configuration space, multiplied by an infinitesimal volume element. In the following, we do not consider the volume element since that is not affected by the confinement, and therefore it will not affect the partition coefficient $\mathcal {W}$ eventually. For clarity, we will use the notation $\tilde{\mathcal {G}}$ to denote the partition function in which the volume element has been neglected.

From $c \le c_1$ it follows $\hat{c}\le c_1$, and therefore the dumbbells cannot see the wall (see Sect. 4.1): For any $\mathbf {Y}$, the entire hypersphere defined by $\hat{c}= c$ is contained in $\varOmega _{X}$. Let $S_N$ denote the surface area of an *N*-dimensional hypersphere of unit radius,

A.3 $$\begin{aligned} S_N = \frac{2 \pi ^{N/2}}{\varGamma (N/2)} \, , \end{aligned}$$

where, here, $\varGamma$ denotes the gamma function used regularly in Mathematics. The volume $V_N$ and amount of surface area $A_N$ of a hypersphere of radius *R* in *N* dimensions are then given by

A.4 $$\begin{aligned} V_N(R)= & {} \int _0^R S_N r^{N-1} dr = (S_N/N) R^N \, , \end{aligned}$$

A.5 $$\begin{aligned} A_N(R)= & {} S_N R^{N-1} \, . \end{aligned}$$

Therefore, the surface area of the hypersphere defined by the condition $\hat{c}= c$ (with $c \le c_1$, i.e. the hypersphere is entirely contained in $\varOmega _{X}$) is given by $A_N(R = \sqrt{N c})$; since this is independent of $\mathbf {Y}$, subsequent integration over $\varOmega _{Y}$ results in

A.6 $$\begin{aligned} \tilde{\mathcal {G}}_0 \propto \left( d_2- d_1\right) ^N \, A_N(R = \sqrt{N c}) \, . \end{aligned}$$

In the following, we consider the case where $\hat{c}$ (and thus *c*) is slightly larger than $c_1$, $\hat{c}\gtrsim c_1$ (“slightly” means that the parts of the hypersphere *not* contained in $\varOmega _{X}$ should be in the form of “caps”); we write $\hat{c}= c_1(1 + \epsilon )$. The amount of surface area of the hypersphere defined by $\hat{c}= c$ which is contained in $\varOmega _{X}$ is given by

A.7 $$\begin{aligned} \tilde{A}_N = A_N - 2 \sum _{\mu =1}^N a_{N,\mu } \, , \end{aligned}$$

where $a_{N,\mu }$ is the surface area of a single cap outside of $\varOmega _{X}$ due to $2 Y_\mu < \sqrt{N c}$, and the factor of 2 accounts for the fact that there are two such caps for each spatial direction (in a completely symmetric way, because the integration domain $\varOmega _{X}$ is completely symmetric w.r.t. $\mathbf {Y}\rightarrow -\mathbf {Y}$). The following should be noted: (i) The condition $d_1\le Y_\mu \le d_2$ together with $2 Y_\mu < \sqrt{N c}$ amounts to the range $d_1\le Y_\mu < d_{1,\epsilon }$ with $d_{1,\epsilon }\equiv d_1\sqrt{1 + \epsilon }$, i.e. the cap-contribution $a_{N,\mu }$ is present only for values of $Y_\mu$ in this narrow range. (ii) The cap surface area (in this limit) does not depend on the values of the other $Y_\nu$ ($\nu \ne \mu$). (iii) Depending on the values of the components of $\mathbf {Y}$, not all caps are “active” at the same time.

For a single cap of a hypersphere in *N* dimensions with radius *R* and intersecting plane at position *Z* from the origin, with $Z \le R$, the volume $v_N$ of this cap is given by

A.8 $$\begin{aligned} v_N = \int _Z^R V_{N-1}(\rho (Z')) dZ' \, , \end{aligned}$$

with $\rho (Z) = \sqrt{R^2 - Z^2}$. The surface area of the cap is then given by

A.9 $$\begin{aligned} a_N= & {} \frac{d v_N}{d R} = S_{N-1} R^{(N-1)} \int _z^1 \left( 1 - z'^2 \right) ^{(N-3)/2} dz' \, , \end{aligned}$$

where we have used $Z = R z$. For the concrete case in question above, we have $R = \sqrt{N c} = 2 d_{1,\epsilon }$ and $Z = 2 Y$ with $2 d_1\le 2 Y < 2 d_{1,\epsilon }$; this implies $z = Z/R = 2 Y / R$ with $1/\sqrt{1+\epsilon } \le z < 1$. This means that the integral in Eq. (A.9) is to be evaluated only for values of *z* that are very close to unity, i.e., we can concentrate on the integrand in the vicinity of $z' = 1$. Using this approximation, one finds eventually for the dominant contribution

A.10 $$\begin{aligned} a_N = \frac{2^{(N-1)/2}}{N-1} S_{N-1} R^{(N-1)/2} \left( R - 2 Y \right) ^{(N-1)/2} \, , \nonumber \\ \end{aligned}$$

where $$R = \sqrt{N c} = 2 d_{1,\epsilon }$$.

For the integration of $\tilde{A}_N$ Eq. (A.7) over the domain $\varOmega _Y$, we note the following: For every one of the *N* dumbbells, the range $[d_1,d_2]$ should be split into two intervals, $[d_1,d_{1,\epsilon }]$ and $[d_{1,\epsilon },d_2]$, where the former of these two is very short, $\varDelta = d_1\left( \sqrt{1+\epsilon }-1\right) \simeq d_1\epsilon /2$ . This subdivision for every dumbbell results in a subdivision of the entire domain $\varOmega _{Y}$: the largest part is $\otimes ^N [d_{1,\epsilon },d_2]$, there are *N* contributions that are linear in $\varDelta$, namely of the form $(\otimes ^{(N-1)} [d_{1,\epsilon },d_2]) \otimes [d_1,d_{1,\epsilon }]$, and there are higher order contributions ($\mathcal {O}(\varDelta ^\beta )$ with $\beta \ge 2$). It is noted that in all parts of $\varOmega _{Y}$ the $A_N$ needs to be counted fully, however for some parts of $\varOmega _Y$ this needs correction, in terms of subtracting contributions related to $a_{N,\mu }$. Integrating $\tilde{A}_N$ Eq. (A.7) (integrating $A_N$ over the entire $\varOmega _Y$, and subtracting the cap-contributions by integration over those parts of $\varOmega _Y$ that are linear in $\varDelta$, i.e., over the dominant non-trivial $\varDelta$-parts of $\varOmega _Y$ which contain only one pair of caps), one obtains

A.11 $$\begin{aligned} \tilde{\mathcal {G}}= & {} (d_2-d_1)^N A_N \nonumber \\&-2 N (d_2-d_{1,\epsilon })^{N-1} \int _{d_1}^{d_{1,\epsilon }} a_{N} dY \, . \end{aligned}$$

With

A.12 $$\begin{aligned} \int _{d_1}^{d_{1,\epsilon }} \left( R - 2 Y \right) ^{(N-1)/2} dY\simeq & {} \frac{1}{N+1}\left( d_1\epsilon \right) ^{(N+1)/2} \, , \nonumber \\ \end{aligned}$$

one obtains

A.13 $$\begin{aligned} \tilde{\mathcal {G}}&\simeq (d_2-d_1)^N A_N \nonumber \\&-\frac{2^{(N+1)/2}}{N^2-1} N S_{N-1} (d_2-d_{1,\epsilon })^{N-1} R^{(N-1)/2} \left( d_1\epsilon \right) ^{(N+1)/2} \, . \end{aligned}$$

This relation can be simplified by using the definition (A.3), which leads to

A.14 $$\begin{aligned} S_{N-1} = \frac{1}{\sqrt{\pi }}\frac{ \varGamma (\frac{N}{2}) }{ \varGamma (\frac{N-1}{2}) } S_N \, , \end{aligned}$$

and therefore, together with (A.5),

A.15 $$\begin{aligned} \tilde{\mathcal {G}}\simeq & {} (d_2-d_1)^N A_N \nonumber \\&-\frac{2^{(N+1)/2}}{N^2-1} N \frac{1}{\sqrt{\pi }}\frac{ \varGamma (\frac{N}{2}) }{ \varGamma (\frac{N-1}{2}) } A_N \nonumber \\&\times (d_2-d_{1,\epsilon })^{N-1} R^{-(N-1)/2} \left( d_1\epsilon \right) ^{(N+1)/2} \, .\nonumber \\ \end{aligned}$$

Since we are only interested in leading order terms in $\epsilon$, one may replace in the last line $d_{1,\epsilon }$ by $d_1$, and *R* by $2 d_1$, which results in

A.16 $$\begin{aligned} \tilde{\mathcal {G}}\simeq & {} (d_2-d_1)^N A_N \nonumber \\&-\frac{2^{-N} }{N^2-1} N^{(N+3)/2} \frac{1}{\sqrt{\pi }}\frac{ \varGamma (\frac{N}{2}) }{ \varGamma (\frac{N-1}{2}) } A_N \nonumber \\&\times (d_2-d_1)^{N-1} d_1^{-N} \left( \delta c \right) ^{(N+1)/2} \, , \end{aligned}$$

where we have made use of

A.17 $$\begin{aligned} \delta c \equiv c - c_1= c_1\epsilon = \frac{4 d_1^2}{N} \epsilon \, . \end{aligned}$$

If we define

A.18 $$\begin{aligned} \gamma _N \equiv \frac{2^{-N} }{N^2-1} N^{(N+3)/2} \frac{1}{\sqrt{\pi }}\frac{ \varGamma (\frac{N}{2}) }{ \varGamma (\frac{N-1}{2}) } \, , \end{aligned}$$

one obtains (tildes can be omitted, since the missing volume elements cancel out)

A.19 $$\begin{aligned} \mathcal {W}- 1= & {} \frac{\mathcal {G}- \mathcal {G}_0}{\mathcal {G}_0} \nonumber \\\simeq & {} - \gamma _N \frac{ 1 }{ d_1^{N} (d_2-d_1) } \left( \delta c \right) ^{(N+1)/2}\, . \end{aligned}$$

In the above treatment it has been assumed that $d_1> 0$. Setting $d_1= 0$, the above treatment does not work, because the cap-scenario no longer holds; for a finite (though small) value of *c*, all cases of intersection of the hypersphere with the hyperrectangle $\varOmega _{X}$ can occur (no part of the sphere contained in $\varOmega _{X}$ for sufficiently small $\mathbf {Y}$; entire sphere contained in $\varOmega _{X}$ for sufficiently large $\mathbf {Y}$; and all cases in between); therefore, the case $d_1= 0$ cannot be treated analytically, and thus there is no analytic prediction for the behavior of $\mathcal {W}$ in this limit.

For the numerical simulations, it is relevant to emphasize the range of validity of the approximation derived in this appendix. Obviously, according to the discussion above, $\epsilon$ must be that small that the parts of the hypersphere outside of the domain $\varOmega _{X}$ are indeed in the form of caps. However, the most stringent condition is implicit in one of the steps behind Eq. (A.12): $\epsilon N \ll 1$, which is respected for the simulation results discussed in Sect. 4.3 for this Limit 1.

## Partition function: Limit 2

In this appendix, the partition function $\mathcal {G}$ is examined for $D=1$ in the limit $c \nearrow c_2$ with $c_2= 4 d_2^2$. Again, as in Appendix A, we will depart from the expressions defined in Eqs. (A.1) and  (A.2). In analogy to Appendix A, we focus on the surface area in configuration space and neglect the volume element, i.e., we examine $\tilde{\mathcal {G}}$ rather than $\mathcal {G}$.

We know that $\mathcal {G}= 0$ for $c > c_2$, see Sect. 4.1. In this appendix, we calculate how this limiting value is approached by *c*-values that are slightly smaller than $c_2$, i.e., we look at the behavior when the (absolute value of) $\delta c = c-c_2$ is small (note: $\delta c$ is negative). It has been shown in the previous appendix that

B.1 $$\begin{aligned} \tilde{\mathcal {G}}_0 = \left( d_2- d_1\right) ^N \, A_N(R = \sqrt{N c}) \, , \end{aligned}$$

if wall effects are *not* taken into account.

In the following, we consider the case where $\hat{c}$ (and thus *c*) is slightly smaller than $c_2$, $\hat{c}\lesssim c_2$ (“slightly” means that the parts of the hypersphere contained in $\varOmega _{X}$ should be in the form of “cornes of a hyperrectangle”); we write $\hat{c}= c_2(1 + \epsilon )$, with negative $\epsilon$. It can be shown that $\hat{c}= c_2(1 + \epsilon )$ implies that each dumbbell $\mu$ must obey

B.2 $$\begin{aligned} X_\mu ^2 \ge \left( 1 + N \epsilon \right) c_2\, , \end{aligned}$$

where it is to be noted again that $\epsilon < 0$; obviously, Eq. (B.2) makes sense only if $\left( 1 + N \epsilon \right) \ge 0$. In the following, it is assumed that $|\epsilon | \ll 1/N$ (also to ensure that the “corners of a hyperrectangle”-assumption still holds). The component $X_\mu$ can therefore take (positive and negative) values that obey the conditions

B.3 $$\begin{aligned} 2 d_{2,\epsilon }\le |X_\mu | \le 2 Y_\mu \, , \end{aligned}$$

with $d_{2,\epsilon }= \sqrt{1 + N \epsilon } d_2$. According to Eq. (B.3) the range of (relevant) $X_\mu$-values is chopped into two parts, $[-2 Y_\mu , 2 Y_\mu ]$ $\rightarrow$ $\varOmega _{X,\mu ,-} \cup \varOmega _{X,\mu ,+}$ which, according to the definition of $\varOmega _{X}$, allows to write $\varOmega _{X}$ as the union of $2^N$ sets,–representative of the $2^N$ “corner-parts” (called “CP” in the following)–,

B.4 $$\begin{aligned} \varOmega _{X} = \bigcup \limits _{\{i_\mu \in \{-,+\}\}}^{\,} \left( \otimes ^{N} \varOmega _{X,\mu ,i_\mu } \right) \, . \end{aligned}$$

Since $\varOmega _{X}$ is invariant w.r.t. $\mathbf {Y}\rightarrow -\mathbf {Y}$, the integral over $\varOmega _{X}$ can be replaced by the integral over the hyperrectangle

B.5 $$\begin{aligned} \varOmega ''_{X} = \otimes ^{N} \varOmega _{X,\mu ,+} = \otimes ^N [2 d_{2,\epsilon }, 2 Y_\mu ]\, , \end{aligned}$$

followed by a multiplication with $2^N$, in order to account for the number of CP.

Looking at the one corner $\varOmega ''_{X}$: In this part, the sphere-surface can be represented by a hyperplane with surface normal ${\varvec{n}}$, in other words, the points $\mathbf {X}$ in the hyperplane must satisfy the condition

B.6 $$\begin{aligned} \sum _\mu X_\mu n_\mu = \zeta \, . \end{aligned}$$

The choice ${\varvec{n}}= (1, 1, 1, \ldots )$ serves as a good approximation for the limiting case examined here, i.e., for the CP defined by $\varOmega ''_{X}$. To determine $\zeta$, one can proceed as follows: Inserting the ansatz $\mathbf {X}= \sqrt{\xi } {\varvec{n}}$ in $\hat{c}= c$ leads to $\xi = c = c_2(1+\epsilon )$. With this, it follows from the condition for the hyperplane, Eq. (B.6), that

B.7 $$\begin{aligned} \zeta = N \sqrt{\xi } = N \sqrt{c} = N \sqrt{c_2(1+\epsilon )} = 2 d_2N \sqrt{1+\epsilon } \, . \nonumber \\ \end{aligned}$$

For given $\mathbf {Y}$, we now need to determine the distance from the actual corner point (for that given $\mathbf {Y}$) to where the hyperplane intersects the hyperrectangle edges that run parallel to the Cartesian axes and run through that corner point. For direction $\nu$, we can do this by using the condition (B.6) with $X_\mu = 2 Y_\mu$ for all $\mu \ne \nu$, and determine the corresponding $X_\nu$,

B.8 $$\begin{aligned} X_\nu = \zeta - 2 \sum _{\mu \ne \nu } Y_\mu \, , \end{aligned}$$

and so the distance $\delta$ of that point to the actual corner is given by

B.9 $$\begin{aligned} \delta \equiv 2 Y_\nu - X_\nu = 2 \left( \sum _{\mu } Y_\mu \right) - \zeta \, , \end{aligned}$$

which does not depend on the direction $\nu$. It is obvious from the definition $\delta \equiv 2 Y_\nu - X_\nu$ and the integration bounds for $X_\nu$ that $\delta \ge 0$. According to [36], the surface area of the part of the hyperplane contained in the hyperrectangle is given by (see also Appendix C)

B.10 $$\begin{aligned} A_{\mathrm{cp},1}= & {} \frac{\sqrt{N}}{(N-1)!} \, \delta ^{N-1} \, , \end{aligned}$$

where the subscript “cp,1” states that this is for a single CP. Therefore,

B.11 $$\begin{aligned} A_{\mathrm{cp}}= & {} \frac{2^N \sqrt{N}}{(N-1)!} \, \delta ^{N-1} \end{aligned}$$

is the sum over all $2^N$ CPs. In order to make the following steps more convenient, we define

B.12 $$\begin{aligned} \phi \equiv \delta /2 = \left( \sum _{\mu } Y_\mu \right) - \zeta /2 \, , \end{aligned}$$

which turns $A_{\mathrm{cp}}$ into

B.13 $$\begin{aligned} A_{\mathrm{cp}}= & {} \frac{\hat{\gamma }_N}{(N-1)!} \, \phi ^{N-1} \, , \end{aligned}$$

with

B.14 $$\begin{aligned} \hat{\gamma }_N \equiv 2^{(2N-1)} \sqrt{N} \, . \end{aligned}$$

We now proceed with the integration over $\varOmega _{Y}$. When considering the hypersphere-condition, the lower bound on $Y_\mu$ is given by Eq. (B.3), i.e. $d_{2,\epsilon }< Y_\mu$, for which it is to be noted that $d_{2,\epsilon }$ is the extreme value of only one of the dumbbells while all others must have $d_2$. As we replace the hypersphere (in the CP) by the hyperplane, this condition changes; in particular, for constant *c* (i.e. constant $\zeta$) one finds that $d_{2,\epsilon }$ needs to be replaced by

B.15 $$\begin{aligned} \tilde{d}_{2,\epsilon }= & {} \frac{\zeta }{2} - (N-1) d_2= d_2\left[ 1 + N \left( \sqrt{1+\epsilon } - 1 \right) \right] \, , \end{aligned}$$

It is straightforward to show that this expression agrees with $d_{2,\epsilon }$ up to and including terms linear in $\epsilon$.

The integration over $\varOmega _{Y}$ is restricted by the condition $\phi \ge 0$, i.e., the integration is to be performed over

B.16 $$\begin{aligned} \varOmega '_{Y}= & {} \left( \otimes ^{N} [\tilde{d}_{2,\epsilon }, d_2]\right) \cap \{\mathbf {Y}\in \mathbb {R}^N: \phi (\mathbf {Y}) \ge 0 \} \, .\nonumber \\ \end{aligned}$$

Therefore, integration over $Y_1$ results in

B.17 $$\begin{aligned} \int _{\tilde{d}_{2,\epsilon }}^{d_2} A_{\mathrm{cp}} dY_1 = \frac{\hat{\gamma }_N}{N!} \left. \phi ^{N} \right| _{Y_1 = d_2} \, . \end{aligned}$$

Performing all other *Y*-integrations, one thus obtains (note: the boundary term from the lower integration-bound vanishes, since $\phi =0$)

B.18 $$\begin{aligned} \tilde{\mathcal {G}}= \frac{\hat{\gamma }_N}{2^{(2N-1)} (2N-1)!} \delta _\mathrm{max}^{2N-1} \, , \end{aligned}$$

with

B.19 $$\begin{aligned} \delta _\mathrm{max}\equiv & {} 2 N d_2- \zeta = N \left( \sqrt{c_2} - \sqrt{c} \right) \, , \end{aligned}$$

which leads to

B.20 $$\begin{aligned} \tilde{\mathcal {G}}= \frac{N^{2N-\frac{1}{2}} }{(2N-1)!} \left( \sqrt{c_2} - \sqrt{c} \right) ^{2N-1} \, . \end{aligned}$$

The dominant-order approximation of this expression can be obtained for the limit when $| \delta c / c_2| \ll 1$; using $\sqrt{c_2} - \sqrt{c} \simeq - \delta c/(4 d_2)$, Eq. (B.20) becomes

B.21 $$\begin{aligned} \tilde{\mathcal {G}}\simeq \check{\gamma }_N \frac{(- \delta c)^{2N-1}}{d_2^{2N-1}} \, , \end{aligned}$$

with

B.22 $$\begin{aligned} \check{\gamma }_N = \frac{ N^{(2N-\frac{1}{2})} }{ 2^{2(2N-1)} (2N-1)! } \, . \end{aligned}$$

For comparing the prediction in Eqs. (B.21) and  (B.22) with the results of the numerical simulations, one can do this without normalizing w.r.t. $\mathcal {G}_0$. However, in this case, one must be careful about the increments, i.e. bins, in the histogram. The bins in the histogram (representative of $\mathcal {G}$) are equally spaced w.r.t. *c*. Therefore, $\tilde{\mathcal {G}}$ above must be multiplied by the increment of the coordinate that is perpendicular to the hyperplane, $\zeta$; this implies the relation

B.23 $$\begin{aligned} \mathcal {G}= \tilde{\mathcal {G}}\, \frac{d \zeta }{dc} \, , \end{aligned}$$

with, according to Eq. (B.7),

B.24 $$\begin{aligned} \frac{d \zeta }{d c}= & {} \frac{N}{2} \frac{1}{\sqrt{c}} \, . \end{aligned}$$

Since we are interested only in the dominant limiting behavior of $\mathcal {G}$ for $c \nearrow c_2$, *c* in the expression on the right-hand side of Eq. (B.24) could also be replaced by $c_2$.

For the numerical simulations, it is mentioned that the prediction in Eq. (B.21) is valid as long as it is reasonable to approximate the intersecting part of the hypersphere by a hyperplane; the range of *c*-values is restricted by the condition $|\epsilon | N \ll 1$, which is respected for the simulation results discussed in Sect. 4.3 for this Limit 2.

## Surface area of hyperplane intersecting a hypercube

Think of the *n*-dimensional hypercube in $\mathbb {R}^n$, described by $0 \le x_i \le L$ for $i=1,\ldots ,n$: $I^n := \otimes ^n [0,L]$. Furthermore, a hyperplane with normal vector ${\varvec{n}}$ parallel to $(1, 1, 1, \ldots )$ is defined by $H_{y}^{n-1} := \left\{ {\varvec{x}} \in \mathbb {R}^n: \sum _{i=1}^n x_i = y \right\}$. In the following, $0 < y \le L$ will be assumed. Note that $H_{y}^{n-1}$ intersects the *i*-axis at position $x_i = y$, which is part of the hypercube.

In the following, we want to calculate the volume $V_n$ of the corner that is chopped-off from the hypercube by the hyperplane. It is straightforward to see that

C.1 $$\begin{aligned} V_n(y) = \int _{0}^{y} V_{n-1}(y-z) dz \, . \end{aligned}$$

This recursive formula can be solved by using the starting value for $n=2$: $V_n(y) = y^2/2$. With this, it can be shown that

C.2 $$\begin{aligned} V_n(y) = \frac{y^n}{n!} \, , \end{aligned}$$

which indeed fulfills Eq. (C.1).

We now proceed with calculating the amount of surface area *A* of the hyperplane $H_{y}^{n-1}$ that is contained in the hypercube $I^n$. It can be shown that

C.3 $$\begin{aligned} A = \frac{d V_n}{d s} \, , \end{aligned}$$

where *s* denotes the coordinate that runs along the normal vector ${\varvec{n}}$ of the hyperplane. Departing from the origin, the normal vector hits the hypersurface at $\tilde{x}_i = y/n$ for all *i*. The distance of this point from the origin is

C.4 $$\begin{aligned} s = \sqrt{\sum _{i=1}^{n} \tilde{x}^2_i} = \frac{y}{\sqrt{n}} \, . \end{aligned}$$

For the area *A*, we thus find

C.5 $$\begin{aligned} A = \frac{d V_n}{d s} = \sqrt{n} \frac{d V_n}{d y} = \frac{\sqrt{n}}{(n-1)!} y^{(n-1)} \, , \end{aligned}$$

which agrees with what can be obtained as a special case of the general expression in [36].

## Numerical calculations

This appendix describes the numerical procedure for calculating the partition function $\mathcal {G}$ and the partition coefficient $\mathcal {W}$, in terms of histograms.

The procedure for the cases $c \searrow c_1$ (limit) and $c_1\le c \le c_2$ (full range) is as follows:

- Subdivide the *c*-range in equally spaced bins; the number of bins is denoted by $n_c$, the number of states by $n_\mathrm{cfg}$. Make a loop over each bin; for each bin, do the following $n_\mathrm{b} \equiv n_\mathrm{cfg}/n_c$ times:
- Choose a vector with components drawn (statistically independently) from a normal distribution, normalize this vector to unit length: this results in a vector from a random (isotopic) distribution *on* the unit hypersphere (in *N* dimensions) (see also [37, 38]).
- For a homogeneous distribution of random points in a hyperspherical shell with radii $R_\mathrm{min}$ and $R_\mathrm{max}$ (corresponding to the minimum and maximum value of *c* of the respective bin), the probability distribution of the length *R* of the homogeneously distributed vectors can be written in the form $p(R) dR = \mathcal {N} N R^{N-1} dR$, where the normalization constant $\mathcal {N}$ is determined by the normalization condition $\int _{R_\mathrm{min}}^{R_\mathrm{max}} p(R) dR = 1$. We aim at sampling this distribution by a quantity *z* that is distributed homogeneously on the interval [0, 1], i.e., $p(R) dR = dz$; integration with lower bound $R = R_\mathrm{min}$ and $z = 0$, respectively, leads to the solution
  D.1 $$\begin{aligned} R = \root N \of {R_\mathrm{min}^N + \left( R_\mathrm{max}^N - R_\mathrm{min}^N \right) z }\, , \end{aligned}$$
  where *z* is drawn from a homogeneous probability distribution on the interval [0, 1].
- For the random vector $\mathbf {X}$ generated in the just-mentioned manner, one (i) includes it in the histogram for $\mathcal {G}_0$ and (ii) includes it also in the histogram for $\mathcal {G}$ if it fulfills $\left| X_\mu \right| \le 2 Y_\mu$ for all $\mu$, where $Y_\mu$ is drawn from a homogeneous distribution on the interval $[d_1,d_2]$.
- The following error-analysis can be done in each bin: Basically, one makes $n_\mathrm{b}$ attempts (which all count towards histogram for $\mathcal {G}_0$) to check whether they are compatible with the wall; the fraction of successful placings (counts towards histogram for $\mathcal {G}$) is given by $\mathcal {W}_\mathrm{b} = n_\mathrm{b,success}/n_\mathrm{b}$. This can be seen as a binary random process of a variable *q* that can take values 0 (failed placing) and 1 (successful placing). With $\mathcal {W}_\mathrm{b} = \langle q \rangle$ the average over all placing attempts, the corresponding variance is given by $\mathrm{var}(q) = \langle \left( q - \mathcal {W}_\mathrm{b} \right) ^2 \rangle$. A straightforward calculation leads to $\mathrm{var}(q) = \mathcal {W}_\mathrm{b} \left( 1 - \mathcal {W}_\mathrm{b}\right)$. Therefore, the standard deviation is given by $\sigma _q = \sqrt{\mathcal {W}_\mathrm{b} \left( 1 - \mathcal {W}_\mathrm{b}\right) }$, from which one can derive the standard error of the mean,
  D.2 $$\begin{aligned} \sigma _{\mathcal {W}_\mathrm{b}} = \sqrt{\frac{\mathcal {W}_\mathrm{b} \left( 1 - \mathcal {W}_\mathrm{b}\right) }{n_\mathrm{b}}} \, . \end{aligned}$$
  In particular, let us consider the case that $W_\mathrm{b}$ is close to zero: Requiring that the standard error of the mean is comparable to the mean, $\sigma _{\mathcal {W}_\mathrm{b}} \simeq \varphi _{\mathcal {W}} \mathcal {W}$ with $\varphi _{\mathcal {W}} \lesssim 1$, one obtains the condition $\mathcal {W}_\mathrm{b} \simeq (\varphi _{\mathcal {W}}^2 n_\mathrm{b})^{-1}$; when fitting the full *c*-range data further below, we make use of $\varphi _{\mathcal {W}} = 1/\sqrt{10}$, which implies $\mathcal {W}_\mathrm{b} \simeq 5 \times 10^{-6}$ because $n_\mathrm{b} = 2 \times 10^{6}$ number of samples per bin; i.e., bins with $\mathcal {W}_\mathrm{b}$ smaller than this value are not used in the fitting, because of too large fluctuations; fitting is done only over the the range that satisfies this condition, and the error is also determined on the basis of this range.

The procedure for the case $c \nearrow c_2$ (limit) is as follows:

- For sampling $\mathbf {X}$ efficiently, we do not want to sample first $\mathbf {X}\in \otimes ^{N} [2 d_{2,\epsilon }, 2 d_2]$ and then reject a large majority of points because they do not satisfy $c_2(1+\epsilon ) \le \hat{c}$. Instead, we want to sample points homogeneously in the intersection of the domains $\varOmega _{X,\epsilon } = \left\{ \mathbf {X}\in \otimes ^{N} [2 d_{2,\epsilon }, 2 d_2]\right\}$ and $\varOmega _\zeta = \left\{ \mathbf {X}\in \mathbb {R}^N: \sum _\mu X_\mu \ge \zeta \right\}$. We choose the value of $\zeta$ such that the plane defined by $\sum _\mu X_\mu = \zeta$ intersects the edges of $\varOmega _\zeta$ at the same points as if $\varOmega _\zeta$ is intersected by the hypersphere defined by $c_2(1+\epsilon ) = \hat{c}$. In analogy to the procedure described in Appendix B, this leads to
  D.3 $$\begin{aligned} \zeta = 2 (N-1) d_2+ 2 d_{2,\epsilon }= 2 d_2\left( N-1+\sqrt{1 + N\epsilon }\right) \, .\nonumber \\ \end{aligned}$$
  In order to select a point from a homogeneous distribution in the intersection of $\varOmega _{X,\epsilon }$ and $\varOmega _\zeta$, we proceed in an iterative way: (i) We determine the minimum possible value for $X_1$: $X_{\min } = \zeta -2(N-1)d_2= 2d_{2,\epsilon }$ (note: for all components, the maximum value is $X_\mathrm{max} = 2d_2$), and (ii) perform the following iterative loop for determining all components $X_\mu$ for $\mu = 1, \ldots , N$:
  - choose $X_\mu$ according to the distribution $p_\mu (X)$;
  - update $$X_\mathrm{min}$$: $$X_\mathrm{min} = X_\mathrm{min} + X_\mathrm{max} - X_\mu $$.
  The distribution $p_\mu (X)$ is given as follows: It must be proportional to the volume $V_{N-\mu }(X-X_\mathrm{min})$ of the hyperrectangle corner (for notation, see Appendix C). Enforcing normalization on the interval $\left[ X_\mathrm{min},X_\mathrm{max}\right]$, one obtains
  D.4 $$\begin{aligned} p_\mu (X) = \frac{N-\mu +1}{\left( X_\mathrm{max}-X_\mathrm{min}\right) ^{N-\mu +1}} \left( X-X_\mathrm{min}\right) ^{N-\mu } \, .\nonumber \\ \end{aligned}$$
  Sampling this distribution with a variable *z* distributed homogeneously on the interval [0, 1], i.e., requiring $p_\mu (X) dX = dz$, one finds after integration the relation
  D.5 $$\begin{aligned} X = X_\mathrm{min} + \left( X_\mathrm{max}-X_\mathrm{min}\right) \, z^{1/(N-\mu +1)} \, . \end{aligned}$$
  After this sampling (which is 100% efficient, i.e., every attempt is successful), one selects only those $\mathbf {X}$ which satisfy $c_2(1+\varepsilon ) \le \hat{c}$ (in this latter step, the efficiency is lower than 100% but still rather high, e.g., 89% for $N=50$).
- The last step consists in taking the condition $X_\mu \le 2 Y_\mu$ (for all $\mu$) into account, where it is noted that we need to examine only one of the corners of the hyperrectangle. A brute-force way to implement this would be to pick homogeneously $\mathbf {Y}\in \otimes ^{N} [d_{2,\epsilon }, d_2]$, and then delete all states $\mathbf {X}$ that do not satisfy the condition; this would result in a drastic decrease of efficiency. A much better way is the following: For given $\mathbf {X}$, we just *imagine* that an $\mathbf {Y}$ was chosen, and we ask for the probability $p_{Y|X}$ that the condition $X_\mu \le 2 Y_\mu$ (for all $\mu$) was fulfilled after having chosen $\mathbf {Y}$; since the $\mathbf {Y}$ is drawn from a homogeneous distribution, $p_{Y|X}$ must be proportional to the volume $\prod _{\mu } \left( X_\mathrm{max} - X_\mu \right)$; we may thus count the generated state $\mathbf {X}$ with a weight proportional to this volume; in this way, there is no additional wasting of generated states (all states that fulfill the condition $c_2(1+\varepsilon ) \le \hat{c}$ contribute to the histogram with this corresponding weight). In the simulations, the weight is normalized in a specific way, to avoid numerical underflow; particularly, the weight is chosen to be the ratio of the above mentioned volume divided by the volume of the chopped-off-corner (intersection of $\varOmega _{X,\epsilon }$ and $\varOmega _\zeta$), and then multiplied by $2^N$, which leads to the weight
  D.6 $$\begin{aligned} \mathrm{weight} = 2^N \, N! \, \prod _\mu \left( \frac{X_\mathrm{max} - X_\mu }{X_\mathrm{max} - X_\mathrm{min}} \right) \, . \end{aligned}$$
- Note: For increasing *N*, the exponent $\beta$ in the limit Eq. (46) can become rather large. Therefore, however wide the range $[c_2(1+\epsilon ),c_2]$ of sampling, the region of highest interest ($c \nearrow c_2$) is sampled quite poorly (the corresponding bins may get just a few hits) in comparison to the region further away from $c_2$. Therefore, if the sampling is insufficient, the exponent $\beta$ is potentially overestimated; in other words: the region $c \nearrow c_2$ is a small corner in the hyperrectangle, and in order to sample that well one needs a substantial increase in sampling points (in the entire domain).

## Declarations

### Data availability statement

This manuscript has associated data in a data repository. [Author’s comment: The datasets generated during and/or analysed during the current study as related to Figs. 5, 6, and 9 are available in the Zenodo repository, https://zenodo.org/deposit/5799710 (see [31] for full details).]
